# The impact of emergency policy (contingency plans) on regional emergency logistics response capacity in China

**DOI:** 10.3389/fpubh.2026.1857393

**Published:** 2026-06-03

**Authors:** Heng Chen, Yanying Wang, Bo Zhou

**Affiliations:** 1 School of Management, Xi’an Polytechnic University, Xi’an, China; 2 School of Economics and Finance of Xian Jiaotong University, Xi’an, China

**Keywords:** difference-in-differences, emergency logistics policy, emergency logistics response capacity, policy consistency, policy effect

## Abstract

**Introduction:**

Emergency policies and contingency plans serve as critical institutional arrangements for enhancing regional emergency response capacity, yet systematic causal evidence regarding their actual effectiveness remains limited. This study aims to empirically assess the impact of emergency policies on emergency logistics response capacity, taking the promulgation of China’s National Master Plan for Public Health Emergency Response in 2006 as a quasi-natural experiment.

**Methods:**

Utilizing panel data from 30 provincial-level administrative regions in China spanning 2005–2022, we employed a staggered difference-in-differences approach. A cosine similarity-based text quantification method was applied to measure policy content consistency between provincial emergency policies and the central master plan. A multidimensional indicator system was constructed to assess regional emergency logistics response capacity, and its relationship with policy consistency was analyzed.

**Results:**

Policy consistency exhibits a foundational supporting effect: low consistency is generally associated with weak response capacity, whereas high consistency alone does not guarantee strong capacity. The implementation of emergency policies led to an average increase in emergency logistics response capacity of 0.0137 units, representing an approximate 47% improvement, a result robust to multiple checks. The policy effect showed a time lag, becoming statistically significant only in the third year post-implementation. Pronounced regional heterogeneity was observed: the effect was significant in eastern China but not in central and western regions; regions with higher policy consistency or higher initial response capacity benefited more substantially.

**Discussion:**

This study advances emergency logistics research by integrating policy text quantification, dynamic capacity decomposition, and causal inference. The findings offer empirical evidence for optimizing regional emergency management policies and fostering cross-regional coordination, with practical insights for developing countries facing analogous challenges. The identified time lag and regional disparities highlight the need for sustained institutional building and differentiated policy strategies.

## 1 Introduction

Amid intensifying global climate change, persistent geopolitical conflicts, and the normalization of public health emergencies, various sudden-onset disasters and crises have exhibited a pattern of “high frequency, wide coverage, severe destructiveness, and compound complexity,” posing significant challenges to regional emergency logistics response capacity worldwide. According to the *Global Humanitarian Logistics Assessment Report* released by the United Nations Office for the Coordination of Humanitarian Affairs (OCHA) in 2024, global economic losses attributable to extreme climate events reached USD 380 billion in 2023, with over 60% of affected areas experiencing exacerbated secondary disasters due to lagging emergency logistics response capacity. Emergency logistics, defined as logistical activities aimed at providing essential supplies for sudden public events—including natural disasters and public health emergencies—with the dual objectives of maximizing event response effectiveness and minimizing disaster losses (1), has a response capacity that directly influences the effectiveness of emergency prevention and control as well as the speed of economic recovery (2). Consequently, emergency logistics response capacity has emerged as a core indicator for assessing national resilience governance. Against this backdrop, establishing an efficient, coordinated, and resilient regional emergency logistics system has become a central lever for enhancing public safety governance capabilities globally. Emergency policies, serving as critical instruments for resource coordination, process standardization, and bottleneck resolution, fundamentally determine the speed, efficiency, and quality of emergency logistics response.

China, as one of the countries most severely affected by natural disasters worldwide, has witnessed frequent occurrences of various emergencies. From the catastrophic Yangtze River floods in 1998 and the Wenchuan earthquake in 2008, to the COVID-19 pandemic in 2020 and the “July 20” torrential rainstorm disaster in Zhengzhou, Henan Province, in 2021, and extending to recent extreme typhoons and forest fires, the challenges confronting China’s regional emergency logistics response capacity have continued to escalate. Nevertheless, after years of development, China has preliminarily established a five-tier emergency material reserve network spanning “central–provincial–municipal–county–township” levels and has promulgated a series of policy documents—including the *National Master Plan for Public Emergency Response* and the *Emergency Response Law of the People’s Republic of China*—aimed at promoting the enhancement of emergency logistics response capacity. Concurrently, various Chinese provinces have formulated emergency-related policies and plans at different stages. This raises a critical question: Have these emergency policies (contingency plans) indeed driven improvements in China’s regional emergency logistics response capacity? Existing research has offered limited insights into this issue.

Internationally, although emergency logistics policies are widely regarded as essential instruments for strengthening emergency response capacity, empirical studies examining the effect of emergency policies (contingency plans) on emergency logistics response capacity remain relatively scarce in the extant literature, with a particular dearth of systematic testing at the provincial level (3, 4). More importantly, prevailing research tends to treat policy interventions as homogeneous, thereby overlooking substantial variations across regions in terms of policy text characteristics, implementation timing, and underlying response capabilities (5). However, policy research has long recognized that the actual effectiveness of policies critically depends on the degree of mutual coordination among policies in terms of their goals, directions, and instruments. In their policy mix design framework, Howlett and Rayner explicitly define policy coherence as the capacity of multiple policy instruments to reinforce rather than offset one another in achieving their respective objectives (6). Pal further refines this concept into three dimensions: internal coherence, external coherence, and temporal coherence (7). The experimental study by van Engen (2019) provides empirical evidence that policy coherence significantly enhances frontline implementers’ perceptions of policy legitimacy (8). In the field of emergency management, vertical policy coherence between central and local governments is particularly critical: the degree of semantic convergence between local emergency plans and the national master plan directly affects the synergistic efficiency of resource mobilization and response speed. However, existing emergency logistics research has rarely quantified policy coherence, let alone incorporated it into a causal inference framework.

Beyond the fact that policy coherence serves merely as a necessary condition for unleashing institutional effectiveness, the enhancement of emergency logistics response capacity also hinges upon cross-organizational and cross-level coordination during policy implementation. In the field of public administration, research on institutional coordination has long demonstrated that cross-sectoral integration mechanisms constitute a critical foundation for policy implementation (9). Against the backdrop of political polarization, coordination difficulties at the local, regional, and national levels further constrain the actual effectiveness of policies (10). Meanwhile, variables such as trust, power-sharing, and leadership style directly influence the performance of cross-agency collaboration (11). At the same time, under sudden shocks such as public health emergencies, the responsiveness of emergency logistics systems is closely intertwined with the resilience of health systems. Early research on health system resilience centered on the system’s absorptive and adaptive capacities, which constitute core dimensions of emergency response (12). Effective shock response depends on the dynamic interaction between the evolutionary characteristics of the shock and system structures across temporal and spatial scales; whether the speed of epidemic transmission matches the response time lag of material reserve networks directly determines the ultimate effectiveness of emergency logistics (13). Furthermore, at the level of crisis governance, how multi-level governance architectures are linked to emergency response outcomes remains insufficiently explored through comparative research (14). The effectiveness of hybrid crisis governance hinges upon the capacity to flexibly recombine control-based and collaborative modes in response to changing constraints (15). Synthesizing these perspectives, the impact of emergency policies on logistics response capacity is not simply linear; rather, it is jointly moderated by policy coherence, institutional coordination capacity, the foundation of system resilience, and crisis governance modes. This study incorporates these theoretical perspectives into its analytical framework to more comprehensively reveal the conditions under which emergency policies can unleash their effectiveness.

As the world’s largest developing country, empirical investigations focusing on the Chinese context provide a unique research setting for examining this relationship. Following the issuance of China’s *National Master Plan for Public Emergency Response* in 2006, Chinese provinces adopted local contingency plans in a staggered manner, generating natural variation in the timing of local implementation under a unified central policy framework. This staggered rollout creates conditions akin to a quasi-experiment, facilitating the identification of the causal effects of emergency logistics policies (16). Moreover, the challenges China faces in constructing its emergency management system exhibit substantial parallels with those encountered by numerous emerging economies. Therefore, research grounded in the Chinese sample holds significant referential value for other nations—particularly developing countries—in scientifically formulating emergency policies and advancing emergency logistics response capacity. Based on the above analysis, this study proposes the following research hypotheses: H1: The implementation of emergency policies can significantly enhance regional emergency logistics response capacity. H2: Policy consistency positively moderates the effect of emergency policies on response capacity, such that the policy effect is stronger in regions with high policy coherence.

Our study aims to empirically test the above hypotheses and elucidate the effects of emergency policies on emergency logistics response capacity. First, we systematically review the emergency plans and policies issued by China’s central government and provincial governments and apply the cosine similarity method to measure the textual coherence between provincial emergency policies and the national master plan, thereby systematically identifying patterns of convergence and divergence. Second, we construct a composite indicator of emergency logistics response capacity, analyze the trends in response capacity across regions, and compare the regular patterns between policy coherence and response capacity. Finally, leveraging the institutional context in which provinces implemented the policy in a staggered manner, we construct a staggered difference-in-differences model to empirically examine the main effect, time-lag characteristics, and regional heterogeneity of emergency policies, as well as the moderating role of policy coherence.

The innovation and contributions of this paper are threefold. First, we operationalize policy coherence from a qualitative concept into a measurable semantic similarity index, thereby overcoming the limitations of word frequency statistics in traditional policy text analysis. Second, we construct a multidimensional emergency logistics response capacity evaluation system encompassing carrier carrying capacity, throughput capacity, velocity capacity, efficiency capacity, informatization capacity, and social emergency support capacity. Third, employing a staggered difference-in-differences (DID) approach for quasi-experimental causal inference, we systematically reveal the time-lag and heterogeneity of policy effects, providing empirical evidence for the formulation of differentiated emergency management policies.

The remainder of this paper is organized as follows. Section 2 presents the literature review and theoretical analysis. Section 3 measures the emergency policy coherence and emergency logistics response capacity of Chinese provinces and conducts a comparative analysis. Section 4 describes the empirical design. Section 5 reports the baseline regression results, robustness checks, and heterogeneity analysis. Section 6 discusses the findings. Section 7 concludes with policy implications.

## 2 Literature review

### 2.1 Research on emergency logistics response capacity

The concept of emergency logistics can be traced back to the 1980s. Dupon explored issues of material allocation and logistical support in disaster response, laying the groundwork for subsequent research (17). Entering the 21st century, the introduction of the humanitarian logistics perspective further enriched this concept. Thomas and Mizushima defined emergency logistics as the entire process of delivering relief supplies and services to affected populations during natural disasters or complex emergencies (18). In China, Ou defined it as logistics activities aimed at providing emergency supplies required for sudden public events, with the goal of minimizing disaster losses (19).

Building on the above definition, emergency logistics response capacity can be further understood as the potential capacity of an emergency logistics system to mobilize, transport, and distribute resources to meet the urgent material needs of disaster-affected areas following an emergency event, with its core attributes being response speed, coverage, and delivery reliability (20). This concept differs from logistics efficiency—which is typically measured by the volume of logistics completed per unit cost, emphasizing the ratio of resource inputs to outputs (21)—and from system resilience—which focuses on a system’s absorptive, adaptive, and transformative capacities after a shock, centering on long-term recovery and dynamic learning (12). In short, response capacity answers the question of “how much can be mobilized and how quickly it can be delivered,” efficiency concerns “whether the cost is reasonable,” while resilience addresses “whether the system can recover from shocks and improve.” This study concentrates on the measurement of response capacity and its policy-driven mechanisms, without directly involving cost efficiency or the long-term evolution of resilience.

Research on the measurement of emergency logistics response capacity has primarily developed along three lines: capability evaluation, performance indicator systems, and supply chain resilience. In terms of capability evaluation, scholars have largely adopted multi-criteria decision-making methods to construct multidimensional indicator systems. For instance, Liu employed a hybrid fuzzy DEMATEL-ISM-MICMAC method to systematically identify key factors affecting the resilience of emergency logistics supply chains, such as infrastructure and plan completeness (22). Lei integrated game theory with the TOPSIS method to evaluate emergency material suppliers from dimensions including emergency resilience and logistics cost, thereby validating the robustness of the combined weighting method (23). In the domain of performance measurement, Anjomshoae conducted a systematic review to map the indicator systems and theoretical lineages of humanitarian supply chain performance measurement from 2007 to 2021 (24). Ahmad, combining fuzzy Delphi and fuzzy DEMATEL, found that quality-dimension indicators such as timely delivery and delivery accuracy play a dominant causal role in the performance of sustainable humanitarian relief logistics (25). In assessing supply chain resilience and flexibility, Chukwuka et al. proposed a fuzzy AHP–fuzzy TOPSIS framework to prioritize mitigation strategies for post-disaster emergency supply chains, identifying collaboration, coordination, and flexible transportation capacity as the most critical strategies (26). Patil and Madaan, using bibliometric and network analysis, delineated seven research clusters in humanitarian supply chains, highlighting performance measurement, sustainability, and innovation as important research frontiers (27). These studies collectively illustrate the evolution of emergency logistics response capacity measurement from single-indicator evaluation toward a deep integration of multidimensional, hybrid methods and resilience perspectives.

### 2.2 Research on emergency policies and emergency logistics response capacity

Emergency policies are widely regarded as an important means of enhancing response capacity. From the perspective of theoretical mechanisms, policies function primarily through three pathways. First, policies promote the informatization of emergency logistics, facilitating information sharing and decision support, thereby improving response speed (28). Second, from a supply chain management perspective, policies play a critical role in coordinating multi-agent actions (29). Third, experience from disaster response practices indicates that coordination mechanisms, resource stockpiling, and information sharing are key factors affecting response capacity (2). Furthermore, recent research based on Chinese provincial panel data has found that the effective organization of government emergency management departments can facilitate the conversion of social logistics into emergency logistics, thereby promoting the enhancement of response capacity (30).

Although the theoretical mechanisms are relatively clear, empirical research on the impact of emergency policies on emergency logistics response capacity remains limited. Comparisons of disaster response practices across multiple countries reveal a significant gap between policy design and implementation capacity (31). A systematic review of the humanitarian logistics field indicates that empirical studies are insufficient in both quantity and depth (32). In fact, the actual effects of emergency logistics policies are often moderated by regional resource endowments and organizational capacity (33). From a broader perspective, further analysis of the humanitarian logistics field corroborates this judgment (34). In recent years, scholars have continued to point out that the existing literature predominantly consists of case studies and qualitative analyses, lacking causal inference based on large-sample data (35). In addition, a few scholars have directly explored the implementation effects of emergency policies. For example, Gao and Wang employed text mining methods to quantitatively analyze local government policy documents and found that differences in policy text content are a key reason for regional disparities in policy implementation outcomes (36).

### 2.3 Research on policy coherence and logistics performance

In recent years, a growing body of research on the causal relationship between policy coherence and logistics performance has gradually delineated a transmission chain running from institutional quality, through reduced uncertainty and strengthened inter-agency coordination, to improved logistics performance. Cross-national evidence indicates that institutional factors centering on economic freedom, the rule of law, and low levels of corruption are key variables determining differences in logistics efficiency across countries (37), and that systematically reforming the institutional and regulatory pillars of the Global Competitiveness Index can significantly enhance logistics performance (38). Regarding transmission mechanisms, uncertainty reduction constitutes a core pathway: trade policy uncertainty generates dynamic spillovers on the shipping industry, ranging from short-term chaos to long-term structural adjustments, and impairs corporate financial performance through two channels—reduced technological innovation and declining supply chain efficiency (39, 40). Another key mechanism lies in strengthening inter-agency coordination. A recent study of member countries of the Central Asia Regional Economic Cooperation program found that clarifying cross-departmental responsibilities and unifying inspection standards through service-level agreements can effectively mitigate the fragmentation of border agencies, thereby enhancing port efficiency and trade facilitation (41). On a comprehensive dimension, government effectiveness has been demonstrated to be the most stable and policy-sensitive predictor of logistics performance; its encompassing capacities for policy formulation and implementation and the quality of public service delivery directly translate into more efficient border clearance and infrastructure execution (42). Moreover, the policy formulation framework driven by the Logistics Performance Index provides countries with a practical tool for moving from performance assessment to policy benchmarking and improvement (43).

Furthermore, the coherence between structural interactions and policy design exerts a dynamic impact on system performance (44). The effectiveness of policy interventions across governance levels depends strongly on their coherence and coordination efficiency (45). Policy coherence and the degree of institutional integration have a significant positive impact on sustainable development performance (46). Effective institutional coordination can enhance the synergistic efficiency and systemic resilience of policy interventions (47). The level of coherence of policy interventions directly affects their effectiveness, while coordination efficiency across governance levels is a key factor determining systemic resilience (48, 49).

### 2.4 Research gaps

A systematic review of existing research reveals that while relevant findings provide important references for the theoretical framework of this study, several notable gaps remain to be addressed. First, although scholars have proposed various definitions and concepts of emergency logistics across different historical periods, there is a lack of a comprehensive indicator system based on these definitions to systematically measure and evaluate emergency logistics response capacity. Second, in terms of measuring emergency logistics response capacity, existing studies have largely focused on performance evaluation of emergency logistics. Given that emergency logistics is a special type of logistics activity that often disregards cost considerations, most research tends to emphasize cost-related metrics while overlooking the most important objective of emergency logistics—namely, minimizing disaster losses. Furthermore, systematic analyses of the temporal evolution and regional disparities in emergency logistics response capacity remain insufficient. Third, with respect to the relationship between emergency policies and emergency logistics response capacity, many scholars have examined the impact of emergency policies from qualitative and practical perspectives. However, empirical research on policy effects is still in its early stages; existing evidence is largely based on case studies or cross-country comparisons, lacking systematic testing and rigorous causal inference using large-sample data. Finally, although a few studies have begun to examine the influence of policy differences on implementation outcomes, and research in the broader field of logistics policy has preliminarily revealed the causal chain linking policy consistency to logistics performance, analyses in the emergency logistics domain remain largely confined to simple statistics of policy keywords or policy intensity. They have failed to conduct systematic quantitative analysis of the deep semantic structures of policy texts, making it difficult to accurately identify the convergence and divergence characteristics of regional emergency policies at the content level—that is, the consistency of emergency policy content.

Considering this, based on existing research findings, this paper makes the following three extensions and innovations. First, in terms of measuring emergency logistics response capacity, drawing on established concepts and definitions of emergency logistics, this study constructs a multi-dimensional comprehensive indicator system that includes potential carrier capacity, potential flow capacity, potential speed capacity, potential efficiency capacity, regional informatization capacity, and social emergency support capacity. It then comprehensively measures the temporal evolution and regional characteristics of emergency logistics response capacity across various regions in China. Second, in terms of emergency logistics policy analysis, this study employs text vectorization and cosine similarity methods to identify the convergence and divergence characteristics of the deep semantic structures of provincial emergency logistics policy texts. Breaking through the limitations of traditional policy text analysis—which often relies on simple keyword frequencies or policy intensity—this approach systematically characterizes interregional policy similarities and differences at the semantic level, thereby laying a solid textual foundation for subsequent heterogeneity analysis of policy effects. Third, in terms of policy effect evaluation, taking advantage of the institutional context in which provinces implemented emergency logistics policies in a staggered manner, this study constructs a staggered difference-in-differences model to empirically test the facilitating effect of emergency logistics policies on regional emergency logistics response capacity. Through a quasi-experimental design, it effectively mitigates policy endogeneity and provides causal inference evidence based on Chinese provincial panel data. Furthermore, it explores the heterogeneous performance of policy effects across different policy characteristics and capacity types, offering a scientific basis for the formulation of differentiated emergency management policies.

## 3 Characteristics of regional emergency policy consistency and emergency logistics response capacity in China

### 3.1 Systematic review of provincial emergency policies in China

Following the Severe Acute Respiratory Syndrome (SARS) epidemic in 2003, China began to place significant emphasis on the formulation of emergency policies (contingency plans) for public health emergencies. The inaugural issuance of emergency-related policy in China occurred in 2006, marked by the promulgation of the National Master Plan for Public Health Emergency Response by the State Council of the People’s Republic of China. Subsequently, provincial authorities across China progressively issued corresponding policies, thereby further refining the national emergency logistics policy framework. This study systematically reviews the emergency-related policies and plans enacted by China’s central government and provincial administrations since 2006, as presented in Table 1.

**Table 1:** Summary of emergency logistics-related policies and plans in China.

Region	Issuance date	Policy (plan)	Main content relate-d to emergency logistics
Central Government of China	January 8, 2006	*National Master Plan for Public Emergency Response*	“Master Plan for Public Emergency Response. The overall emergency plan serves as the overarching framework for the national emergency response plan system and constitutes the normative document for the State Council’s response to extraordinarily serious public emergencies.”
Shanxi Province	January 18, 2006	*Implementation Opinions of the People’s Government of Shanxi Province on Promoting the Development of the Circulation Industry*	“…Establish a reserve system for essential commodities and an emergency control system… Provincial-level competent commerce authorities shall designate a group of key enterprises linked for emergency commodities. In exceptional circumstances requiring urgent deployment of emergency goods, key enterprises must produce, sell, and transport them as required… Each municipality shall also establish corresponding reserve and emergency control systems…”
Tianjin Municipality	February 9, 2006	*Notice on Issuing the Outline of the Eleventh Five-Year Plan for National Economic and Social Development of Tianjin Municipality*	“Establish and improve emergency response mechanisms for public emergencies to ensure the safety of the New Area. Enhance the earthquake monitoring and early warning system and strengthen rapid response capabilities.”
Anhui Province	April 29, 2006	*Notice of the People’s Government of Anhui Province on Issuing the Opinions on Key Work Arrangements for 2006*	“…Vigorously develop logistics distribution and chain operations and cultivate a group of provincial-level key circulation enterprises. Strengthen the construction of emergency management systems and capabilities, explore the establishment of an emergency management mechanism featuring unified command, comprehensive functions, sensitive response, and efficient operation. Compile the provincial Eleventh Five-Year Plan for Emergency Management Construction, study the initiation of a provincial emergency management information platform, and strengthen the development of professional emergency management teams…”
Sichuan Province	January 19, 2007	*Notice of the General Office of the People’s Government of Sichuan Province on Issuing the ‘Eleventh Five-Year’ Plan for the Development of Modern Logistics Industry in Sichuan Province*	“…Construct an emergency logistics system. Designate a group of warehousing, transportation, and integrated logistics enterprises as national, provincial, and municipal-level emergency logistics enterprises to undertake critical logistics tasks during emergencies. Strengthen coordination between local logistics and military logistics resources to enhance support functions and promote the construction of the emergency logistics system…”
Gansu Province	April 13, 2007	*Notice of the General Office of the People’s Government of Gansu Province on Issuing the Guiding Opinions on Economic System Reform Work for 2007 in Gansu Province*	“…Establish and improve emergency management systems and mechanisms to enhance emergency response capabilities…”
Guangdong Province	July 23, 2007	*Notice of the General Office of the People’s Government of Guangdong Province on Issuing the ‘Eleventh Five-Year’ Plan for Disaster Prevention and Reduction in Guangdong Province*	“…Strengthen the construction of the earthquake forecasting system… Implement emergency funds and material reserves…”
Shanghai Municipality	August 2, 2007	*“Eleventh Five-Year’”Plan for National Economic and Social Informatization of Shanghai Municipality*	“…Based on the city’s Eleventh Five-Year Plan for the Construction of the Emergency Response System for Public Emergencies, construct and improve an emergency management information platform addressing natural disasters, accidents, public health events, and social security incidents. Refine the municipal emergency linkage information platform and further integrate the foundational information platform for comprehensive urban disaster reduction…”
Heilongjiang Province	August 17, 2007	*Outline of the Medium- and Long-Term Science and Technology Development Plan of Heilongjiang Province (2006–2020)*	“…In the field of public safety, enhance technical support for rapid response and emergency disposal of major public safety incidents. Establish a technical system for scientific prediction, effective prevention and control, and efficient emergency response. Improve early detection and prevention capabilities and enhance comprehensive emergency rescue capacity. Focus on fundamental, critical, and generic technologies such as emergency information systems for incident prevention, assessment, and disposal, as well as monitoring and defense against major natural disasters…”
Inner Mongolia Autonomous Region	May 19, 2008	*Notice of the People’s Government of Inner Mongolia Autonomous Region on Issuing the Key Work Points for 2008*	“…Further strengthen emergency management efforts. Enhance emergency management training and public science education to improve society-wide capabilities for preventing and responding to emergencies… Actively develop the modern logistics industry and elevate the level of specialized and socialized logistics services…”
Beijing Municipality	May 19, 2008	*Implementation Opinions of the People’s Government of Beijing Municipality on Further Strengthening Surveying and Mapping Work*	“…Establish and improve emergency management surveying and mapping support mechanisms to provide timely and reliable geographic information and technical services for major events and the prevention and disposal of public emergencies…”
Zhejiang Province	February 18, 2009	*2009 National Economic and Social Development Plan of Zhejiang Province*	“…Conduct investigations of potential safety hazards in production and carry out special rectification campaigns in key industries and sectors to prevent the occurrence of major accidents. Improve emergency response mechanisms for public emergencies, advance disaster prevention and reduction projects such as embankment reinforcement and housing fortification, strengthen grassroots emergency management, and enhance emergency response capabilities…”
Shandong Province	May 8, 2009	*Notice of the People’s Government of Shandong Province on Issuing the Revitalization and Development Plan for the Modern Logistics Industry of Shandong Province*	“…Establish information systems for emergency production, circulation, transportation, and logistics enterprises to facilitate urgent deployment during emergencies. Establish a multi-level government emergency material reserve system to meet the needs of emergency regulation. Strengthen the construction of emergency logistics facilities, equipment, and technical support platforms to enhance emergency response capabilities. Vigorously integrate emergency logistics resources, and select and cultivate a group of logistics enterprises with emergency response capabilities to establish an emergency logistics system…”
Jiangsu Province	May 27, 2009	*Outline of the Logistics Industry Adjustment and Revitalization Plan of Jiangsu Province*	“…Gradually establish an emergency logistics linkage mechanism. Select and cultivate a group of logistics enterprises with emergency response capabilities, establish an emergency logistics system, and enhance the capacity to respond to emergencies such as disasters and major epidemics…”
Hebei Province	June 11, 2009	*“Eleventh Five-Year” Plan for National Economic and Social Informatization of Hebei Province*	“…Construct provincial and municipal-level work safety emergency rescue command centers and specialized emergency rescue systems. Establish a disaster emergency relief command system…”
Jilin Province	September 4, 2009	*Notice on Issuing the Guiding Opinions on Strengthening Industrial Emergency Management Work*	“…Improve the industrial emergency management work system. The Ministry of Industry and Information Technology is responsible for national industrial emergency management work and undertakes the disposal of relevant national emergencies as stipulated… Establish an emergency linkage mechanism for industrial product support. Improve the mechanism for the emergency deployment of industrial products and actively promote the construction of the logistics system for emergency industrial products…”
Guizhou Province	November 8, 2009	*Work Plan for Implementing the Logistics Industry Adjustment and Revitalization Plan in Guizhou Province*	“…Formulate the ‘Special Plan for Emergency Logistics in Guizhou Province’… Establish information systems for emergency production, circulation, transportation, and logistics enterprises to facilitate urgent deployment during emergencies… Strengthen the construction of the emergency logistics system and implement emergency logistics projects. Enhance the capacity to respond to emergencies such as disasters and major epidemics…”
Guangxi Zhuang Autonomous Region	January 20, 2010	*Notice of the People’s Government of Guangxi Zhuang Autonomous Region on Issuing the Plan for Logistics Industry Adjustment and Revitalization in Guangxi*	“…Based on the autonomous region’s emergency response system, establish an emergency logistics system. Integrate social resources for emergency production, processing, storage, and transportation. Strengthen the construction of emergency logistics infrastructure and improve the efficiency of emergency logistics transportation. Municipalities and relevant industry management departments shall establish emergency material reserve systems and emergency information systems, formulate emergency logistics plans, and enhance capabilities for responding to emergencies…”
Hubei Province	July 30, 2010	*Notice of the People’s Government of Hubei Province on Issuing the ‘Twelfth Five-Year’ Development Plan for E-Government in Hubei Province*	“…Strengthen the construction of emergency platforms for provincial, municipal, and county-level governments. Improve specialized emergency management systems in key industries and sectors to achieve organic linkage between government emergency platforms at all levels and departmental emergency management systems. Enhance emergency response capabilities to ensure that emergency reactions and responses are agile, orderly, and efficient…”
Qinghai Province	August 3, 2010	*Notice of the General Office of the People’s Government of Qinghai Province on Issuing Policies and Measures to Accelerate the Development of the Logistics Industry in Our Province*	“The management of emergency plans follows the principles of comprehensive coordination, classified management, hierarchical responsibility, and primary local jurisdiction.”
Fujian Province	September 30, 2010	*Regulations of Fujian Province on Promoting the Development of the Modern Logistics Industry*	“…Strengthen the construction of emergency logistics facilities and equipment, select and cultivate a group of logistics enterprises with emergency response capabilities, establish an emergency logistics system, and enhance emergency response capabilities…”
Hunan Province	July 7, 2011	*“Twelfth Five-Year” Development Plan for the Petrochemical Industry of Hunan Province*	“…Improve the emergency logistics system, enhance the modernization, standardization, and safety level of chemical logistics, and prevent the occurrence of major chemical safety accidents…”
Shaanxi Province	August 3, 2011	*Opinions of the General Office of the People’s Government of Shaanxi Province on Further Strengthening the Construction of the Emergency Material Support System*	“…Fully leverage the favorable opportunities presented by the province’s rapid economic development and industrial transformation and upgrading, accelerate the establishment of an emergency industry platform, establish an emergency industry system, and promote the development of the emergency industry…”
Jiangxi Province	April 9, 2014	*Notice of Jiangxi Province on the Implementation Opinions for Further Promoting the Healthy and Rapid Development of the Modern Logistics Industry*	“…Encourage the accelerated development of logistics in key areas. Encourage and support the modern logistics industry in utilizing high-tech means, promoting energy conservation and emission reduction, and developing green logistics. Strengthen the construction of the emergency logistics system and enhance the level of emergency logistics support…”
Henan Province	November 11, 2015	*Opinions of the General Office of the People’s Government of Henan Province on Accelerating the Development of the Emergency Industry*	“…Focus on developing services such as emergency logistics, traffic rescue, flood fighting and rescue, drought relief and reduction, emergency medical rescue, safety risk assessment, monitoring and early warning, work safety, fire safety, environmental monitoring, security engineering, hazard investigation, emergency management market consulting, engineering rescue, and emergency insurance…”
Hainan Province	December 1, 2015	*Notice of the People’s Government of Hainan Province on Issuing the Implementation Plan for the Development of the Modern Logistics Industry during the ‘Thirteenth Five-Year’ Period in Hainan Province*	“…In accordance with the principles of dual-use for military and civilian purposes, peacetime-wartime integration, and integration of combat readiness, disaster relief, and emergency response, accelerate the establishment of a unified, coordinated, rapid-response, orderly, efficient, and reliable emergency logistics system to enhance logistics emergency support capabilities…”
Yunnan Province	March 17, 2016	*Implementation Opinions of the General Office of the People’s Government of Yunnan Province on Accelerating the Development of the Emergency Industry*	“…Further improve the construction of emergency monitoring and emergency dispatch command systems. Build a comprehensive information platform for emergency products and production capacity reserves to enhance cross-departmental, cross-regional, and cross-industry collaborative support and information sharing capabilities for emergency materials…”
Chongqing Municipality	April 22, 2017	*Notice of the General Office of the People’s Government of Chongqing Municipality on Issuing the ‘Thirteenth Five-Year’ Plan for the Construction of the Emergency Response System for Public Emergencies in Chongqing*	“…Establish and improve the emergency logistics system. Strengthen the standardization of emergency logistics and establish and refine relevant standards and specifications for emergency logistics…”
Liaoning Province	October 23, 2018	*Implementation Opinions of the People’s Government of Liaoning Province on Vigorously Developing the Modern Logistics Industry*	“…Promote the development of emergency logistics. Focus on responding to natural disasters and serving national defense needs. Improve emergency support plans, strengthen emergency logistics coordination and command, and coordinate the collection, transportation, scheduling, and distribution of emergency rescue materials to ensure the rapid, orderly, precise, and efficient operation of the emergency logistics system…”
Ningxia Hui Autonomous Region	October 11, 2021	*“Fourteenth Five-Year” Plan for the Development of Modern Logistics in Ningxia Hui Autonomous Region*	“…Improve the trunk transportation and branch radiation network for emergency logistics in our region. Enhance the organizational level for large-scale urgent deployment of materials and strengthen emergency logistics support capabilities for addressing various types and levels of emergencies…”
Xinjiang Uygur Autonomous Region	August 15, 2022	*“Fourteenth Five-Year” Plan for the Development of Modern Logistics Industry in Xinjiang Uygur Autonomous Region*	“…Strengthen the construction of the emergency logistics facility network… Improve the emergency logistics support mechanism… Optimize emergency logistics organization…”

### 3.2 Measurement of policy consistency of provincial emergency policies in China

In this paper, policy coherence refers to the capacity of multiple policy instruments to reinforce rather than offset one another in achieving their respective objectives (6). Within the context of emergency management, this concept can be further operationalized as the degree to which provincial emergency policies and the national master emergency plan maintain coordination and consistency in goal setting, directional orientation, instruments, and temporal alignment.

To translate the above definition into a measurable analytical framework, this paper draws on theories of policy coordination and institutional coherence to decompose emergency policy coherence into four dimensions:

First, goal coherence. It examines whether provincial sub-goals align with the national overall strategy, avoiding departmentalism and goal fragmentation, and ensuring that multiple goals reinforce rather than conflict with one another. In a multi-level governance system, crisis governance performance does not depend solely on institutional forms, but more on the institutional capacity constituted by administrative infrastructure, intergovernmental forums, and policy learning capabilities, as well as a political culture that incentivizes collaboration—when a clear national direction is effectively coupled with empowered local implementation, intergovernmental forums can transform overlapping responsibilities into compensatory actions (50). Thus, goal coherence constitutes the core link coupling national direction with local implementation: the goal alignment between provincial policies and the national master plan forms the institutional prerequisite for the effective functioning of the emergency logistics system.

Second, directional and orientation coherence. Horizontally, it requires that policies of functional departments such as finance, health, and transportation work in the same direction; vertically, it requires the vertical integration of policies from the central down to the provincial, municipal, and county levels, while maintaining internal consistency of value orientations such as equity, safety, and sustainability, thereby avoiding fragmented and contradictory policies issued by different departments. The effectiveness of intergovernmental coordination is highly dependent on the institutional context: in multi-level systems with a high degree of decentralization, insufficient coordination often leads to a decline in crisis response performance, and the impact of coordination on policy effectiveness varies significantly across different types of multi-level systems (51). This implies that directional and orientation coherence is not simply a matter of policy alignment; rather, it requires embedding effective coordination mechanisms within horizontal interdepartmental collaboration and vertical hierarchical integration to prevent the fragmentation of policy directions.

Third, instrument and means coherence. It emphasizes that various emergency policy instruments—such as fiscal subsidies, material reserves, tax reductions, and regulatory measures—should be functionally complementary and unified in rules and standards, forming a synergistic force in achieving their respective direct objectives and avoiding offsetting effects. This is precisely the concentrated embodiment of the capacity to “reinforce rather than offset one another.” The fragmentation of institutional frameworks and insufficient coordination among stakeholders constitute core obstacles to post-disaster recovery; when top-level decisions are disconnected from grassroots needs and policy instruments lack functional integration, a coordination failure that is “locally rational but globally detrimental” emerges (52). Thus, instrument and means coherence requires that emergency policy instruments achieve unified rules and functional complementarity at the institutional design level, so as to ensure that multiple instruments truly reinforce one another in the process of achieving their respective objectives.

Fourth, temporal coherence. It requires that core emergency guidelines remain stable and continuous, with old and new plans seamlessly connected, undergoing dynamic fine-tuning as circumstances change while keeping the core orientation unchanged, so as to prevent abrupt shifts or policy volatility. In multi-level polities, although decentralization may exacerbate policy inconsistency, intergovernmental coordination can “amplify” the advantages of decentralization—regions with better coordination not only exhibit more consistent policies and clearer crisis communication but also possess stronger intertemporal policy alignment capabilities (53). This logic directly underpins the institutional foundation of temporal coherence: policy stability and continuity are not isolated technical requirements but are embedded within the institutional capacity for multi-level coordination.

Based on the above framework, this paper further examines the coherence between provincial emergency policies, plans, or contingency plans and the national master emergency plan. Since the National Master Emergency Plan for Public Health Emergencies serves as an overarching guideline, the extent to which provinces can operationalize the goals, orientations, instruments, and temporal requirements embedded therein directly represents the degree of policy implementation and affects regional emergency logistics response capacity. Therefore, to further investigate the policy coherence between Chinese provincial emergency policies, plans, or contingency plans and the national master emergency plan, this paper determines emergency policy coherence through a textual content analysis of the National Master Emergency Plan for Public Health Emergencies and the emergency policies, plans, or contingency plans of all provinces nationwide. Taking the characteristic word frequencies and semantics of the four dimensions as the constituent elements of text vectors, this paper characterizes the coherence between each province’s emergency policies, plans, or contingency plans and the National Master Emergency Plan for Public Health Emergencies by calculating the cosine similarity between the vectors representing the emergency content descriptions in the national plan and those in each provincial document. The cosine similarity calculation method is detailed as follows shown in Equations 1–3:

The essence of text similarity calculation involves first converting two text segments into numerical vectors of equal dimensionality and subsequently applying the cosine similarity formula to measure the cosine of the angle between the two vectors. A value approaching 1 indicates greater similarity in semantic content and/or term frequency distribution between the texts.

Let the n-dimensional feature vectors corresponding to the two text segments be denoted as follows:


$$\vec{A}=(a_{1},a_{2},\ldots a_{n}),\vec{B}=(b_{1},b_{2},\ldots b_{n})$$ (1)

The cosine similarity formula is presented as follows:


$$\cos\theta =\frac{\vec{A}\vec{\cdot B}}{\left\|\vec{A}\|\cdot \|\vec{B}\right\|}$$ (2)

Wherein, the expansion of $\left\|\vec{A}\|,\|\vec{B}\right\|$ is calculated as follows:


$$\|\vec{A}\|=\sqrt{a_{1}^{2}+a_{2}^{2}+a_{3}^{2}\cdots a_{n}^{2}},\|\vec{B}\|=\sqrt{b_{1}^{2}+b_{2}^{2}+b_{3}^{2}\cdots b_{n}^{2}}$$ (3)

In the formula, $\vec{A}$ and $\vec{B}$ represent two n-dimensional vectors, which must be of identical dimensionality; $\vec{A}\vec{\cdot B}$ denotes the inner product of the vectors; θ represents the angle between the vectors; cosθ denotes the resulting cosine function value; and $\left\|\vec{A}\|,\|\vec{B}\right\|$ represent the magnitudes of the respective vectors. A larger value of the resulting index cosθ indicates stronger policy consistency, whereas a smaller value indicates weaker consistency.

### 3.3 Measurement of emergency logistics response capacity in China

#### 3.3.1 Constituent elements of emergency logistics response capacity

Emergency logistics constitutes a specialized form of logistical activity aimed at providing urgent support for the demand of materials, personnel, and funds in response to public emergencies, including severe natural disasters, sudden public health events, industrial accidents, public security incidents, and military conflicts. Like conventional logistics, emergency logistics is comprised of fundamental elements such as fluid carriers, flow direction, flow volume, flow process, and flow velocity (19). It represents a distinct category of logistical activity derived from general logistics operations, undertaken to maximize time efficiency and overall effectiveness while minimizing losses, and specifically oriented toward meeting the material demands of emergency-affected locations. Emergency logistics response capacity, in turn, refers to the potential comprehensive capability exhibited throughout the entire logistics supply process under the impact of public emergencies. This encompasses latent capacities across multiple dimensions—including response time, response speed, quality of emergency material transportation, timeliness of emergency material delivery, and delivery reliability—all mobilized to mitigate disaster impacts and satisfy the urgent material requirements arising in the aftermath of emergencies.

In empirical research concerning logistics capability and emergency logistics capability, a considerable body of scholarship has reached a degree of consensus regarding the measurement indicators for logistics carrier carrying capacity, flow volume capacity, and flow velocity capacity. Building upon the elemental framework, and drawing on the conceptual definition of emergency logistics, its constituent elements, and the methodologies employed in existing studies, this paper recognizes that conventional commercial logistics essentially underpins the formation of emergency logistics response capacity. Accordingly, this study categorizes the constituent factors of emergency logistics response capacity into the following indicators: potential carrying capacity of emergency logistics carriers, potential flow volume capacity of emergency logistics, potential flow velocity capacity of emergency logistics, and potential flow efficiency capacity of emergency logistics. Furthermore, considering that the safeguarding of public medical resources under the impact of public emergencies is primarily borne by governments at various levels, relevant indicators of potential social emergency support capacity are incorporated as additional constituent factors of emergency logistics response capacity. Additionally, given that regional informatization capability constitutes a critical factor influencing the speed and adaptability of emergency logistics response capacity, this dimension is further introduced as a constituent element. Regarding sample data selection, and constrained by the actual availability of data, the sample observed in this study comprises 30 provincial-level administrative regions in China (excluding Tibet Autonomous Region and the special administrative regions of Hong Kong, Macao, and Taiwan). The temporal span of the sample observations extends from 2005 to 2022. All data utilized are sourced from the *China Statistical Yearbook* and the official website of the National Bureau of Statistics of China. Certain indicators are derived through calculations based on raw data, such as the proportion of fixed asset investment in the logistics industry relative to total social fixed asset investment. For individual instances of missing data, imputation methods or analogical estimation techniques are employed to substitute the missing values. The specific composition of the indicator system is presented in Table 2.

**Table 2:** Constituent factors and indicator system of emergency logistics response capacity.

Primary indicator	Secondary indicator	Tertiary indicator	Indicator attribute
Carrying capacity of emergency logistics carriers	Logistics infrastructure	Transport network density	+
	Logistics transport equipment ownership	Civil vehicle ownership	+
		Highway commercial vehicle ownership	+
	Logistics industry employment	Employment in railways, highways, aviation, loading/unloading and other transport, warehousing, and postal services	+
	Logistics carrier volume	Express delivery volume	+
Flow volume capacity of emergency logistics	Freight volume	Railway freight volume	+
		Highway freight volume	+
	Freight turnover	Railway freight turnover	+
		Highway freight turnover	+
Flow velocity capacity of emergency logistics	Proportion of classified highways	Proportion of expressways	+
		Proportion of first-class highways	+
		Proportion of second-class highways	+
Flow efficiency capacity of emergency logistics	Logistics volume per unit of labor	Ratio of freight volume to employment	+
		Ratio of freight turnover to employment	+
	Logistics volume per unit of capital investment	Ratio of freight volume to logistics capital investment	+
		Ratio of freight turnover to logistics capital investment	+
Regional informatization capacity	Telecommunications business volume	Total volume of postal and telecommunication services	+
	Network infrastructure	Length of optical fiber cable lines	+
	Regional communication level	Number of mobile phone subscribers	+
		Number of internet broadband access ports	+
Social emergency support capacity	Rescue support foundation	Number of centers for disease control and prevention	+
		Number of health technical personnel	+
		Number of beds in health institutions	+
	Regional policy support	Proportion of local fiscal expenditure on transportation relative to total fiscal expenditure	+

#### 3.3.2 Method for measuring emergency logistics response capacity

In accordance with the objectives of this study and taking into consideration the respective strengths and limitations of various quantitative evaluation methods, this paper employs the entropy-weighted TOPSIS method to measure emergency logistics response capacity across China for the period spanning 2005 to 2022. The entropy-weighted TOPSIS method first determines indicator weights based on the entropy weight method and subsequently calculates the Euclidean distance of each evaluation object from the positive ideal solution to derive the relative closeness coefficient. This closeness coefficient serves as the basis for ranking and evaluation. The TOPSIS method integrated with entropy weighting enables an objective reflection of practical issues and facilitates the efficient processing of panel data, rendering it well-suited for the measurement of emergency logistics response capacity in this study. The specific computational procedure of this method is outlined as follows shown in Equations 4–10:

1. Standardization of raw data. To facilitate the comparison of indicators across different provinces and years, the data are subjected to dimensionless standardization, ultimately yielding measurement values for the emergency logistics response capacity of each provincial region in China. Given that all indicators selected in this study are positive indicators, the data processing formula is expressed as follows:

$$x_{\mathrm{\theta ij}}^{\prime }=\frac{x_{\mathrm{\theta ij}}-\min\{x_{\theta 1j},\ldots ,x_{\mathrm{\theta nj}}\}}{\max\{x_{\theta 1j},\ldots ,x_{\mathrm{\theta nj}}\}-\min\{x_{\theta 1j},\ldots ,x_{\mathrm{\theta nj}}\}}$$ (4)

Wherein: assuming r years, n provincial-level administrative regions (provinces, autonomous regions, or municipalities), and m indicators, let $x_{\mathrm{\theta ij}}$ denote the value of the j-th indicator for province i in year *θ*, where θ=1,2,…,r;j=1,2,…,n;j=1,2,…,m . $x_{\mathrm{\theta ij}}$ represents the original value of the indicator; $x_{\max}$ and $x_{\min}$ denote the maximum and minimum values, respectively, within the group to which the indicator belongs; and $x_{\mathrm{\theta ij}}^{\prime }$ represents the standardized value of the indicator.

2. Entropy weight calculation.

(1) Compute the proportion $P_{\mathrm{\theta ij}}$ of the i-th alternative under the j-th indicator relative to the total sum of that indicator:

$$P_{\mathrm{\theta ij}}=\frac{x_{\mathrm{\theta ij}}^{\prime }}{\sum_{\theta =1}^{r}\sum_{i=1}^{n}x_{\mathrm{\theta ij}}^{\prime }}$$ (5)

(2) Calculate the information entropy $e_{j}$ for the j-th indicator:

$$e_{j}=-\frac{\sum_{\theta =1}^{r}\sum_{i=1}^{n}({p_{\mathrm{\theta ij}}}^{*}\ln p_{\mathrm{\theta ij}})}{\ln\mathrm{rn}}$$ (6)

(3) Determine the weight $w_{j}$ for the j-th indicator:

$$w_{j}=\frac{1-e_{j}}{\sum_{j=1}^{n}(1-e_{j})}$$ (7)

3.Construction of weighted decision matrix Z.

$$\begin{array}{l} Z={\left(z_{\mathrm{\theta ij}}\right)}_{r\times m\times n},Z_{\mathrm{\theta ij}}=\\ w_{j}\times x_{\mathrm{\theta ij}}^{\prime }(\theta =1,2,\ldots ,r,j=1,2,\ldots ,n,j=1,2,\ldots ,m) \end{array}$$

4. Subsequently, the Euclidean distances from each alternative to the positive ideal solution $d_{j}^{+}$ and the negative ideal solution $d_{j}^{-}$ are computed. The development level of the digital economy for province, denoted as $C_{\mathrm{\theta i}}$ , is then calculated as:

$$D_{\mathrm{\theta i}}^{+}=\sqrt{\sum_{j-1}^{n}{\left(d_{j}^{+}-z_{\mathrm{\theta ij}}\right)}^{2}},d_{j}^{+}=\max(z_{\mathrm{\theta ij}})$$ (8)

$$D_{\mathrm{\theta i}}^{-}=\sqrt{\sum_{j=1}^{n}{\left(d_{j}^{-}-z_{\mathrm{\theta ij}}\right)}^{2}},d_{j}^{-}=\min(z_{\mathrm{\theta ij}})$$ (9)

5. Calculation of emergency logistics response capacity $C_{\mathrm{\theta i}}$

$$C_{\mathrm{\theta i}}=\frac{D_{\mathrm{\theta i}}^{-}}{D_{\mathrm{\theta i}}^{+}+D_{\mathrm{\theta i}}^{-}}$$ (10)

The value of $C_{\mathrm{\theta i}}$ ranges between 0 and 1, where values approaching 1 indicate a relatively advanced emergency logistics response capacity, and values near 0 suggest underdeveloped emergency logistics response capacity.

### 3.4 Analysis of measurement results

#### 3.4.1 Measurement of regional emergency policy consistency in China

The policy consistency between provincial emergency policies, plans, or contingency plans and the national master emergency plan was measured using the cosine similarity calculation method. The results are presented in Table 3. Examination of the measured values reveals a substantial regional disparity in policy consistency, ranging from a minimum of 0.15 for Tianjin to a maximum of 0.8945 for Chongqing. The mean policy similarity across the 30 provinces is approximately 0.530, indicating a moderate-to-high overall level, yet accompanied by pronounced differentiation. The consistency with which localities align with the national contingency plan is notably uneven, exhibiting a distinct stratified gradient. Furthermore, this study employs a three-tier hierarchical clustering approach to classify the sample data, partitioning the sample into regions characterized by high policy consistency (measurement value ≥ 0.75), moderate policy consistency (measurement value between 0.50 and 0.74), and low policy consistency (measurement value < 0.50).

**Table 3:** Consistency between provincial emergency policies, plans, or contingency plans and the national master plan by region in China.

Region	Province	Policy/plan consistency level
East China Mean Policy Consistency: 0.53006	Beijing	0.38
	Tianjin	0.15
	Hebei	0.41
	Shanghai	0.6517
	Jiangsu	0.48
	Zhejiang	0.6289
	Fujian	0.45
	Shandong	0.68
	Guangdong	0.82
	Hainan	0.65
Central China Mean Policy Consistency: 0.5023	Shanxi	0.18
	Jilin	0.82
	Heilongjiang	0.52
	Liaoning	0.52
	Anhui	0.68
	Jiangxi	0.3157
	Henan	0.78
	Hubei	0.48
	Hunan	0.21
West China Mean Policy Consistency: 0.5549	Sichuan	0.2986
	Inner Mongolia	0.7832
	Guizhou	0.53
	Yunnan	0.6873
	Shaanxi	0.79
	Gansu	0.61
	Qinghai	0.1863
	Ningxia	0.57
	Chongqing	0.8945
	Xinjiang	0.2745
	Guangxi	0.48

The high policy consistency group comprises Chongqing, Jilin, Guangdong, Shaanxi, Inner Mongolia, and Henan. A common characteristic among these regions is that they are predominantly provinces with high incidences of mountainous disasters, meteorological disasters, or geological hazards, or are densely populated or economically pivotal provinces. These regions adhere rigorously to the national emergency top-level framework, undertaking minimal localized revisions, thereby exhibiting exceptionally strong consistency in textual content, structural architecture, and procedural alignment.

The moderate policy consistency group includes Shanghai, Zhejiang, Anhui, Shandong, Yunnan, Gansu, Liaoning, Heilongjiang, Guizhou, Ningxia, and other provinces. In these regions, the foundational framework of contingency plans and policies conforms to the national template, albeit with moderate differentiation and optimization tailored to regional considerations such as urban safety, watershed governance, border stability maintenance, and industrial security.

The low policy consistency group encompasses the provinces and centrally-administered municipalities of Tianjin, Shanxi, Qinghai, Hunan, Xinjiang, Sichuan, Jiangxi, Beijing, Hebei, Fujian, Jiangsu, Hubei, and Guangxi. The emergence of this pattern may be attributable to several factors. For municipalities such as Tianjin and Beijing, mature autonomous emergency governance systems with numerous locally distinctive mechanisms have resulted in deviation from the generic national paradigm. In remote northwestern provinces such as Qinghai and Xinjiang, unique geographic conditions and disaster typologies necessitate a high degree of policy customization. Central inland provinces like Hunan and Shanxi tend to reconstruct contingency plans based on localized risk profiles, resulting in low adherence to the national foundational template. Notably, economically advanced provinces such as Jiangsu, Hubei, and Fujian also exhibit low policy consistency, suggesting that economic development is not synonymous with high policy alignment; rather, consistency appears contingent upon the degree of autonomy in local emergency governance pathways.

From a geospatial perspective, regions exhibiting high policy consistency are predominantly concentrated in the southwestern and northwestern areas of China. Specifically, policy consistency measurement values for Chongqing, Shaanxi, Inner Mongolia, and Yunnan cluster at higher levels. These regions are characterized by a high incidence of geological disasters, suggesting that provincial authorities therein may have implemented mandatory alignment with unified national emergency policy standards. In contrast, North China exhibits a concentration of lower consistency values. Notably, Tianjin and Shanxi display relatively low policy consistency, while Beijing and Hebei fall within the intermediate-to-weak range, indicating pronounced localization characteristics in the contingency plans of the Beijing-Tianjin-Hebei region. The measurement values for provinces in South China and Northeast China exhibit marked polarization: Guangdong and Jilin demonstrate exceptionally high policy consistency, whereas the remaining northeastern provinces maintain moderate levels. Provinces within the Yangtze River Delta region display a moderate and stable pattern, with Shanghai and Zhejiang exhibiting relatively high consistency and Jiangsu showing lower consistency, thereby indicating a lack of uniformity in the implementation standards of policy consistency within this sub-region.

#### 3.4.2 Measurement of regional emergency logistics response capacity in China

Based on the aforementioned indicator system, this study employs Stata software and applies the entropy-weighted TOPSIS method to calculate the emergency logistics response capacity of 30 provinces (municipalities and autonomous regions) in China for the period spanning 2005 to 2022. The measurement results are presented in Table 4. To comprehensively capture the developmental dynamics of regional emergency logistics response capacity, and in accordance with the regional classification methodology adopted by the National Development and Reform Commission of China, the Chinese provincial-level regions are categorized into three major economic regions—East, Central, and West. The measurement results for these three economic regions are subsequently analyzed.

**Table 4:** Mean values of emergency logistics response capacity by province in China.

Region	Province	Mean value
East China Mean Policy Consistency: 0.53006	Beijing	0.0442
	Tianjin	0.0114
	Hebei	0.0365
	Shanghai	0.037
	Jiangsu	0.04
	Zhejiang	0.034
	Fujian	0.0197
	Shandong	0.0537
	Guangdong	0.073
	Hainan	0.0078
Central China Mean Policy Consistency: 0.5023	Shanxi	0.035
	Jilin	0.0113
	Heilongjiang	0.0116
	Liaoning	0.0327
	Anhui	0.04
	Jiangxi	0.0234
	Henan	0.045
	Hubei	0.0775
	Hunan	0.0311
West China Mean Policy Consistency: 0.5549	Sichuan	0.0371
	Inner Mongolia	0.0298
	Guizhou	0.0128
	Yunnan	0.02
	Shaanxi	0.026
	Gansu	0.0097
	Qinghai	0.0021
	Ningxia	0.0049
	Chongqing	0.0236
	Xinjiang	0.0144
	Guangxi	0.0235

**Figure 1:**
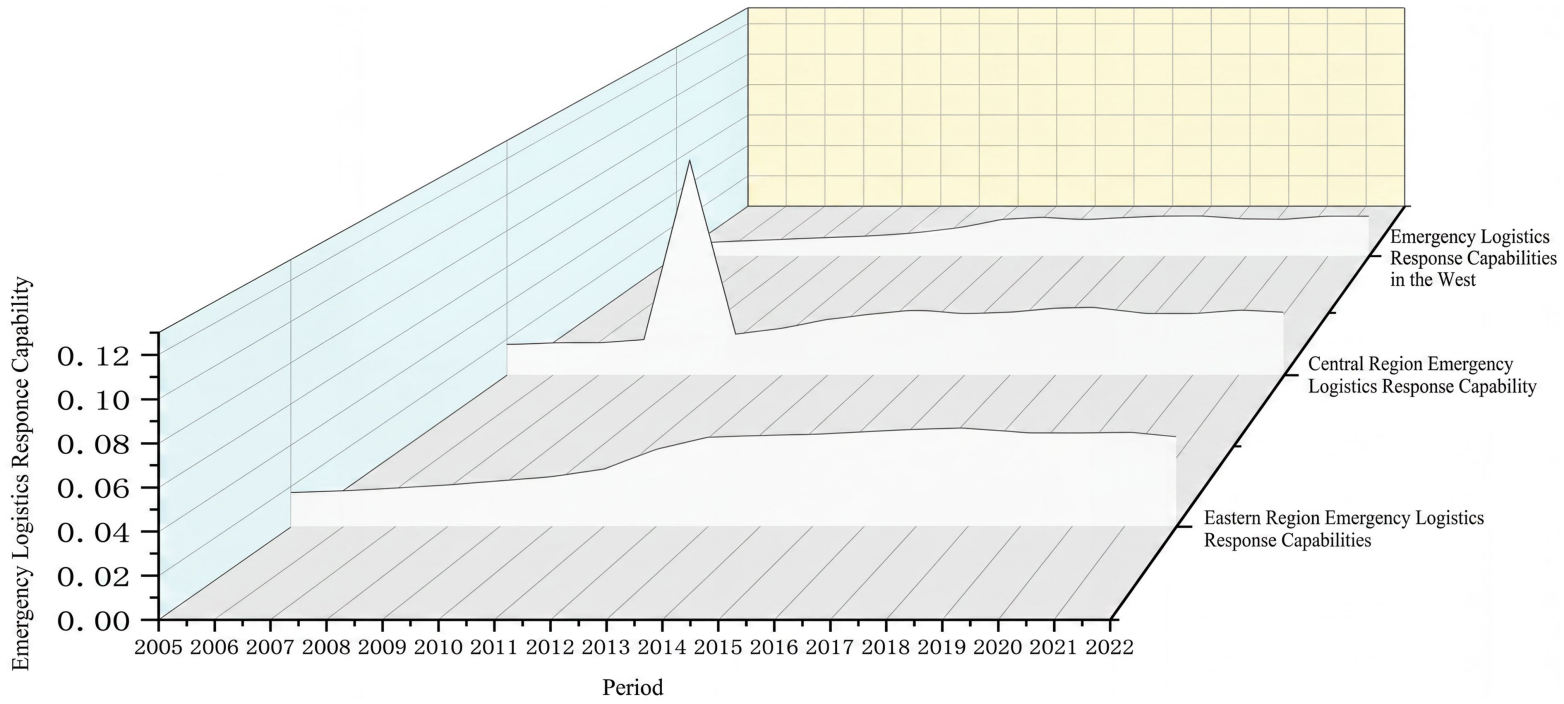
Trends in emergency logistics response capacity across regions in China.

Furthermore, this study analyzes the temporal trends of regional emergency logistics response capacity in China (as illustrated in Figure 1). At the national level, emergency logistics response capacity in the eastern, central, and western regions of China exhibited a consistent upward trajectory during the period from 2005 to 2022. The overall level of national emergency logistics response capacity in China demonstrated an increasing trend; however, during the 2019–2022 period, potentially attributable to the impact of the COVID-19 pandemic, emergency logistics response capacity gradually declined.

From a regional perspective and with respect to the observed trends, emergency logistics response capacity in the eastern, central, and western regions of China all exhibited a steady upward trajectory. The level of emergency logistics response capacity followed a descending gradient from the eastern region to the central region and further to the western region (East > Central > West). Both the eastern and central regions-maintained emergency logistics response capacity levels above the national average, whereas the western region consistently registered a relatively lower capacity, remaining below the national average throughout the period. Furthermore, in terms of the magnitude of improvement, the average growth rate in the eastern region reached 6.07%, followed by the western region at 4.15%, and the central region at 3.39%. Overall, the enhancement of emergency logistics response capacity in China exhibits pronounced regional heterogeneity, with the eastern region demonstrating a higher growth rate than the western region, and the western region, in turn, exhibiting a higher growth rate than the central region.

Observed at the provincial level (as shown in Table 5), the mean values of emergency logistics response capacity across Chinese provinces for the period 2005–2022 permit their classification into four distinct tiers, thereby reflecting hierarchical disparities in emergency logistics response capacity. The high-level tier (≥0.050) comprises only three provinces—Hubei, Guangdong, and Shandong—whose mean values significantly exceed the national average. Hubei registers the national peak value, indicating that these provinces possess notable advantages in emergency material dispatch, logistics network responsiveness, and cross-regional coordination, and thus constitute core supporting regions within the national emergency logistics system. The medium-high level tier (0.035–0.0499) includes Henan, Beijing, Jiangsu, Anhui, Hebei, Sichuan, Shanghai, and Shanxi. This tier predominantly consists of economically developed municipalities and provinces, populous provinces, or major transportation hubs, characterized by well-established emergency logistics infrastructure and robust organizational systems, with response capacity positioned in the upper echelon nationally. The medium level tier (0.020–0.0349) encompasses Zhejiang, Liaoning, Hunan, Inner Mongolia, Shaanxi, Chongqing, Guangxi, Jiangxi, Fujian, Yunnan, and other provinces, whose mean values approximate or fall slightly below the national average. While the emergency logistics response capacity of these provinces generally meets the requirements for regional emergency incident management, a discernible gap persists relative to high-level provinces, and overall performance remains stable and balanced. The low-level tier (<0.020) primarily consists of Qinghai, Ningxia, Hainan, Gansu, Jilin, Tianjin, Heilongjiang, Guizhou, Xinjiang, and analogous regions, with mean values markedly lower than the national average. Qinghai records the lowest value nationwide. These provinces are largely situated in remote western or northeastern border areas, where emergency logistics response capacity remains comparatively weak owing to constraints imposed by geographic location, economic foundation, and transportation conditions (Table 6).

**Table 5:** Measurement results of regional emergency logistics response capacity in China.

Year	East China	Central China	West China
2005	0.0167	0.017	0.009
2006	0.0177	0.018	0.009
2007	0.0191	0.018	0.010
2008	0.0206	0.020	0.011
2009	0.0226	0.119	0.012
2010	0.0246	0.023	0.013
2011	0.0283	0.026	0.015
2012	0.0378	0.031	0.018
2013	0.0438	0.034	0.023
2014	0.0447	0.036	0.024
2015	0.0452	0.034	0.023
2016	0.0464	0.035	0.024
2017	0.0477	0.037	0.025
2018	0.0479	0.037	0.025
2019	0.0462	0.034	0.023
2020	0.0457	0.034	0.023
2021	0.0462	0.036	0.025
2022	0.0442	0.035	0.025

**Table 6:** Patterns of policy consistency and emergency logistics response capacity.

Category	Provinces
High Policy Consistency + High Emergency Logistics Response Capacity	Guangdong, Shanghai, Anhui, Shandong, Henan, Beijing
Low Policy Consistency + Low Emergency Logistics Response Capacity	Tianjin, Shanxi, Guangxi, Xinjiang, Fujian, Hainan, Jiangxi, Guizhou, Yunnan, Gansu, Qinghai, Ningxia

#### 3.4.3 Relationship between regional emergency policy consistency and emergency logistics response capacity in China

A comparative examination of the relationship between emergency policy consistency and emergency logistics response capacity across Chinese provinces reveals, from a correlational perspective, a weak positive correlation between policy consistency and emergency logistics performance within the study sample. Computation yields a Pearson correlation coefficient of 0.1831 between the two variables, indicating that policy consistency exerts a modest positive influence on emergency logistics response capacity.

From the perspective of categorical statistical results, a total of 12 provinces exhibit dual low performance in terms of both low policy consistency (≤0.525) and low emergency logistics response capacity (≤0.0279), accounting for 40% of the total sample. Over 40 % of the regions manifest a dual deficiency characterized by “low policy consistency—weak emergency logistics response capacity,” suggesting that insufficient policy consistency within the sample significantly constrains the enhancement of emergency logistics capacity. Nevertheless, high policy consistency (>0.525) does not invariably engender high emergency logistics response capacity. Only six provinces concurrently demonstrate both high policy consistency and high emergency logistics response capacity, representing merely 20% of the total sample. Several provinces with high policy consistency—including Chongqing, Jilin, and Inner Mongolia—nonetheless maintain emergency logistics performance at intermediate or lower levels.

From the perspective of regional distribution, western and border provinces such as Qinghai, Ningxia, Gansu, and Xinjiang generally exhibit dual low characteristics in terms of both policy consistency within the sample and emergency logistics performance. In such regions, the degree of policy consistency is low and coordination is poor; concurrently, constrained by factors including remote geographic location, resource scarcity, and weak logistics infrastructure, overall emergency support capacity remains relatively feeble. Conversely, eastern coastal provinces and central hub provinces (such as Guangdong, Shandong, and Hubei) demonstrate comparatively superior performance in both policy consistency within the sample and emergency logistics response capacity. In summary, policy consistency within the sample exerts a foundational supporting effect on regional emergency logistics response capacity. Low policy consistency is generally accompanied by low emergency logistics response capacity; however, high policy consistency does not directly determine high emergency logistics response capacity.

## 4 Empirical examination of the impact of emergency policies on regional emergency logistics response capacity in China

The preceding analysis reveals a weak positive correlation between provincial emergency policy consistency and emergency logistics response capacity in China and further suggests that insufficient emergency policy consistency may significantly constrain the enhancement of emergency logistics capacity. To further verify the actual impact of emergency policies and policy consistency on emergency logistics response capacity, this section conducts the following empirical examination.

### 4.1 Variable selection and description

1. Dependent variable: emergency logistics response capacity (*C*). In accordance with the research objectives of this study, emergency logistics response capacity is selected as the core dependent variable and is measured using the entropy-weighted TOPSIS method. The data are sourced from the *China Statistical Yearbook*, with the sample observation period spanning the years 2005–2022.
2. Core explanatory variable: *DID*, namely the implementation of emergency logistics policies (contingency plans).
3. Control Variables:

(1) Number of forest fires (lnforest). Emergency logistics policies aim to enhance response capacity through optimized resource allocation and transportation scheduling. However, forest fires, as high-frequency sudden-onset disasters, continuously deplete the reserve resources and response forces of the emergency logistics system (54). A single forest fire necessitates the mobilization of substantial firefighting personnel, suppression equipment, and logistical supplies; high-frequency occurrences further induce “response fatigue,” thereby weakening the system’s capacity to address other types of emergencies (55). If a policy implementation region coincides with a high-incidence fire zone, any observed decline in response capacity may be erroneously attributed to policy failure. Conversely, capacity enhancements in low-incidence fire zones may be overestimated. Therefore, incorporating the number of forest fires allows for the isolation of resource-depleting pressures stemming from disaster frequency, thereby enabling a more accurate identification of the net effect of policies on response capacity. The sample observation period spans from 2005 to 2022, and the data are sourced from the China Environment Yearbook.
(2) Total area of fire sites (lnarea). The effectiveness of emergency logistics policies hinges upon the unimpeded flow of logistics networks and the sufficiency of transport capacity. The total area of fire sites directly reflects the scale and severity of forest fire disasters: larger fire areas correspond to more extensive fireline spread, significantly increasing the transportation distance and distribution complexity of relief supplies, thereby imposing greater strain on the sustained supply capacity of emergency logistics (56). Failing to control for the area of fire sites could result in observed differences in policy effects being attributable to disparities in disaster magnitude rather than the genuine impact of policy intervention. The inclusion of this variable allows for the elimination of foundational capacity depletion caused by disaster intensity, thereby yielding a more precise estimate of the policy coefficient. The sample observation period spans from 2005 to 2022, and the data are sourced from the China Environment Yearbook.
(3) Number of geological disasters (*lndisaster*). Geological disasters (e.g., landslides, debris flows) exert a unique physical impact on emergency logistics systems. Compared to other types of disasters, geological hazards frequently cause direct damage to roads and bridges, thereby obstructing the transportation corridors for emergency supplies and constraining logistics accessibility and delivery timeliness (57). Such impacts are independent of policy interventions and are characterized by suddenness and spatial unpredictability. If left uncontrolled, policy estimates may be confounded by the “road network failure” effect induced by geological conditions, thereby failing to accurately reflect the role of policies themselves in clearing, rerouting, or restoring transportation networks. The inclusion of this variable facilitates the isolation of structural disturbances arising from geological disasters. The sample observation period spans from 2005 to 2022, and the data are sourced from the *China Environment Yearbook*.
(4) Regional economic development level (lngdp). The effectiveness of emergency logistics policy implementation is highly contingent upon regional logistics infrastructure and fiscal support capacity. Regions with higher levels of economic development typically possess denser transportation networks, more advanced warehousing facilities, stronger information technology capabilities, and more ample fiscal resources. These endowments inherently enhance the foundational capacity of emergency logistics, thereby generating an “economic dividend.” Concurrently, improvements in regional emergency logistics response capacity contribute to promoting the sustainability of economic growth, indicating a bidirectional interactive relationship between the two (58). If left uncontrolled, estimates of policy effects would be confounded by the capacity endowment effects stemming from long-term economic foundations. The inclusion of GDP allows for the isolation of the systematic influence of economic development level on emergency logistics response capacity, thereby enabling the policy coefficient to more accurately reflect the marginal contribution of institutional intervention. The sample observation period spans from 2005 to 2022, and the data are sourced from the China Statistical Yearbook.

### 4.2 Model specification

Given that the issuance of emergency logistics policies (contingency plans) occurred in a staggered sequence across provinces, this study constructs a staggered difference-in-differences (DID) model. The specific OLS effect model is specified as follows shown in Equation 11:


$$\begin{array}{l} C_{i,t}=\alpha_{o}+\alpha_{1}\mathrm{DI}D_{i,t}+\alpha_{2}\text{Infores}t_{i,t}+\alpha_{3}\text{Inare}a_{i,t}+\\ \alpha_{4}\text{Indisaste}r_{i,t}+\alpha_{5}\text{Ingd}p_{i,t}+\varepsilon_{i,t} \end{array}$$ (11)

In the regression model specified above, C denotes the dependent variable, emergency logistics response capacity; DID represents the core explanatory variable, namely the implementation of emergency logistics policies (contingency plans). Specifically, when a given region issues an emergency logistics policy (contingency plan) in a particular year, the value of this explanatory variable takes the value of 1 for that region in the year of issuance and all subsequent years, and 0 otherwise. Lnforest denotes the number of forest fires; lnarea denotes the total area of fire sites; lndisaster denotes the number of geological disasters; lngdp denotes the level of regional economic development; i indexes region; t indexes year; and $\varepsilon_{\mathrm{it}}$ denotes the random error term.

### 4.3 Descriptive statistics

Descriptive statistics for the main variables are presented in this section to reveal the fundamental characteristics of the sample data. The results are shown in Table 7.

**Table 7:** Descriptive statistics.

VarName	Obs	Mean	Median	SD	Min	Max
C	540	0.0292	0.0250	0.0419	0.0010	0.8980
DID	540	0.6093	1.0000	0.4884	0.0000	1.0000
Lnforest	540	3.8516	3.9415	1.7415	0.0000	8.5279
Lnarea	540	5.7914	6.0626	2.5436	0.0000	12.9369
Lndisaster	540	3.7220	3.5835	2.4239	0.0000	11.2771
Lngdp	540	10.5421	10.6032	0.6743	8.5599	12.1547

The dependent variable, emergency logistics response capacity, exhibits a mean value of 0.0292, a median of 0.0250, a standard deviation of 0.0419, and a range spanning from 0.0010 to 0.8980. The relatively low overall mean suggests that, during the sample period, the emergency logistics response capacity across provinces remained at a generally modest level. Meanwhile, the substantial disparity between the maximum and minimum values indicates pronounced regional imbalances, with certain provinces—potentially benefiting from more developed logistics infrastructure or more effective policy implementation—demonstrating considerably higher emergency logistics capacity.

The core explanatory variable, DID, exhibits a mean value of 0.6093, a median of 1.0000, a standard deviation of 0.4884, and a range spanning from 0 to 1. Given that this variable constitutes a dummy indicator for policy implementation, its mean directly reflects the policy coverage ratio, indicating that approximately 60.93% of the provincial-level observations within the sample period correspond to provinces that had issued and implemented emergency logistics policies. This suggests relatively extensive policy diffusion; however, the persistence of provinces yet to implement such policies provides a control group essential for identifying the policy effect.

With respect to the control variables, lnforest exhibits a mean of 3.8516, a median of 3.9415, a standard deviation of 1.7415, and a range spanning from 0.0000 to 8.5279. The substantial degree of variation in the number of forest fires indicates that certain provinces experience frequent fire occurrences while others encounter them only rarely, a pattern primarily attributable to differences in natural environmental and climatic conditions. Lnarea displays a mean of 5.7914, a median of 6.0626, a standard deviation of 2.5436, and a range spanning from 0.0000 to 12.9369. The dispersion of the total area of fire sites is similarly pronounced, reflecting considerable disparities in the scale of impact exerted by forest fires across different regions. Lndisaster records a mean of 3.7220, a median of 3.5835, a standard deviation of 2.4239, and a range spanning from 0.0000 to 11.2771. The overall frequency of geological disasters remains relatively low; however, certain regions characterized by complex geological conditions or insufficient preventive measures exhibit markedly higher occurrence rates. Lngdp presents a mean of 10.5421, a median of 10.6032, a standard deviation of 0.6743, and a range spanning from 8.5599 to 12.1547. Disparities in economic development level across provinces are comparatively modest, yet a discernible gradient persists. Such unevenness may influence both the regional distribution of emergency logistics response capacity and the effective realization of policy effects.

### 4.4 Correlation analysis

Table 8 reports the correlation coefficients among the main variables. A significant positive correlation exists between emergency logistics response capacity (C) and the core explanatory variable DID, which preliminarily confirms that the implementation of emergency logistics policies is positively associated with the enhancement of regional response capacity, consistent with the theoretical expectation of this paper. From an institutional perspective, this result implies that provinces implementing the policy may have obtained institutional support earlier in emergency response, thereby exhibiting a relative advantage in response capacity.

**Table 8:** Correlation analysis.

Variables	C	DID	Lnforest	Lnarea	Lndisaster	lngdp
C	1.000					
DID	0.129^***^	1.000				
	(0.003)					
Lnforest	0.002	−0.242^***^	1.000			
	(0.958)	(0.000)				
Lnarea	−0.033	−0.148^***^	0.144^***^	1.000		
	(0.447)	(0.001)	(0.001)			
lndisaster	−0.019	−0.086^**^	0.247^***^	0.014	1.000	
	(0.667)	(0.046)	(0.000)	(0.751)		
lngdp	0.005	−0.362^***^	0.141^***^	0.054	0.068	1.000
	(0.910)	(0.000)	(0.001)	(0.207)	(0.117)	

* *p*<0.1, ** *p*<0.05, *** *p*<0.01.

No significant correlation is observed between response capacity and any of the control variables. This finding is not surprising: emergency logistics response capacity is a composite indicator whose level is not simply determined by any single factor such as the frequency of natural disasters or the scale of the economy. In other words, a sound economic foundation or high disaster exposure alone does not automatically translate into emergency response capability; what truly matters are institutionalized policy arrangements and coordination mechanisms. This also underscores, from a lateral perspective, the necessity of this paper’s focus on the effects of emergency logistics policies.

Furthermore, significant negative correlations of varying degrees exist between DID and the control variables. This reflects the structural characteristics of the regional distribution of policy implementation timing: regions with relatively lower levels of economic development or relatively smaller disaster risks may exhibit different patterns in the timing of policy rollout. This phenomenon suggests that it is necessary to control for these factors in the subsequent regression analysis in order to more accurately isolate the net effect of the policy itself. Overall, the correlation coefficients among the variables remain at low levels, and no obvious signs of multicollinearity are detected, which provides a foundation for the reliability of the multivariate regression analysis.

### 4.5 Endogeneity test

To mitigate potential endogeneity concerns, this paper lags all control variables by one period and conducts an endogeneity test. The results are presented in Table 9.

**Table 9:** Two-stage least squares (2SLS) results using lagged DID as instrument.

Variable	Coefficient	Std. Err	*t*-value	*p*-value
DID (lagged one period)	3.784***	0.386	9.81	0.000
Lnforest	−0.001***	0.000	−2.95	0.003
Lnarea	0.000***	0.000	−3.04	0.002
Lndisaster	0.000***	0.000	−0.98	0.325
Lngdp	−4.856***	0.179	−27.08	0.000
Constant	2062.485***	2.044	1009.02	0.000
Summary statistics	Value			
R-squared	0.732			
Number of observations	539			
Chi-square	2502.87(*p* = 0.000)			

* *p*<0.1, ** *p*<0.05, *** *p*<0.01.

To test whether the DID model suffers from endogeneity bias, we adopt the lagged term of the policy implementation timing as an instrumental variable and perform a two-stage least squares (2SLS) regression. The first-stage regression results show that the F-statistic for the instrumental variable on the endogenous explanatory variable is 358.165 (*p* = 0.0000), well above the 10% critical value of the Stock–Yogo weak instrument test (16.38), indicating the absence of a weak instrument problem and that the instrumental variable satisfies the relevance condition. The second-stage endogeneity test (a Hausman-type test) yields a *p*-value of 0.6507, far exceeding 0.05, failing to reject the null hypothesis that the explanatory variable is exogenous. This suggests that the baseline DID model does not suffer from significant endogeneity issues. In terms of economic significance, the estimated DID coefficient is 3.784, while the sample mean of the dependent variable is 2013.5 and its standard deviation is 5.185. The policy effect accounts for approximately 0.19% of the dependent variable mean, but its absolute magnitude represents 73% of the dependent variable’s standard deviation, indicating that the effect constitutes a substantial share of data variation and that its statistical significance rests on a solid empirical basis. In sum, both the instrumental variable test and the endogeneity test support the exogeneity assumption of the DID model, confirming that the baseline OLS model is a valid and appropriate estimation method.

Weak instrument test: First-stage F-statistic = 358.17 (*p* = 0.000), rejecting the null of weak instrument.

Endogeneity test: Robust score chi^2^(1) = 0.205 (*p* = 0.651), failing to reject exogeneity; no severe endogeneity is found.

## 5 Analysis of empirical results

### 5.1 Baseline regression results

This paper employs an OLS regression model to examine the impact of emergency logistics policies (DID) on emergency logistics response capacity under the shock of public emergencies. The regression results are presented in Table 10. Column (1) includes only the core explanatory variable DID, while column (2) further incorporates control variables, including the number of forest fires, total burned area, the number of geological disasters, and the level of regional economic development.

**Table 10:** Baseline regression analysis.

	Emergency logistics response capacity	
	(1)	(2)
	C	C
DID	0.0114^*^	0.0137^**^
	(1.9362)	(2.4431)
Lnforest		0.0000
		(0.6604)
Lnarea		0.0000
		(−0.8469)
Lndisaster		−0.0000**
		(−0.9025)
Lngdp		0.0036
		(1.2497)
_cons	0.0216^***^	−0.0180
	(3.9798)	(−0.5826)
*N*	540	540
*F*	3.7488^*^	2.9981 ^*^
r2_a	0.0147	0.0121

t statistics in parentheses. ^*^p < 0.1, ^**^p < 0.05, ^***^p < 0.01.

In column (1), the estimated DID coefficient is 0.0114, statistically significant at the 10% level. Given that the mean emergency logistics response capacity during the sample period is 0.0292, this implies that provinces that implemented the policy exhibited approximately 39% higher response capacity than those that did not. This suggests that, in the absence of the policy, the emergency logistics capacity of these provinces would have reached only about 70% of the current level. After including control variables in column (2), the DID coefficient rises to 0.0137, significant at the 5% level, corresponding to a capacity improvement of approximately 47%. The increase in the coefficient indicates that, after excluding the confounding effects of natural disaster factors and economic development levels, the net effect of the policy itself becomes more pronounced. The coefficients of all control variables are statistically insignificant, a finding that further underscores the necessity of emergency logistics policies as institutionalized intervention instruments.

From the perspective of model explanatory power, the adjusted R^2^ values in columns (1) and (2) are 0.0147 and 0.0121, respectively, indicating relatively low levels. This implies that the model explains only approximately 1.2–1.5% of the variation in emergency logistics response capacity. However, this is because emergency logistics response capacity is a composite indicator whose variation is influenced by a large number of structural factors that are difficult to observe. Specifically, systematic public data are lacking on the level of emergency material reserve infrastructure across provinces; the emergency management organizational capacity and cross-departmental coordination efficiency of local governments represent soft institutional factors that are difficult to quantify; and geographic and climatic conditions also systematically affect disaster response speed. Nonetheless, the low R^2^ does not undermine the reliability of the core findings. In the presence of substantial unobservable heterogeneity, the DID coefficient remains statistically significant with a robust sign, indicating that the policy effect exhibits considerable resilience. Furthermore, if omitted variables are uncorrelated with the policy variable, they do not cause estimation bias; if they are correlated, the estimated coefficient is typically attenuated toward zero, suggesting that the value of 0.0137 obtained in this paper may represent a relatively conservative lower bound. Taken together, although the model does not exhaust all influencing factors, the positive net effect of emergency logistics policies can be confirmed. Provinces that implemented the policy exhibit approximately 40–47% higher response capacity than those that did not, a magnitude corresponding to tangible improvements in rescue efficiency. Therefore, the theoretical hypothesis H1 of this paper is supported: emergency logistics policies effectively improve regional emergency logistics service capacity through mechanisms such as optimizing resource allocation and enhancing transportation efficiency.

### 5.2 Parallel trend test

When employing the difference-in-differences (DID) model for policy effect evaluation, satisfying the parallel trend assumption constitutes a critical prerequisite for ensuring unbiased estimation results. This assumption requires that, prior to policy implementation, the treatment group and the control group exhibit identical trends in the core dependent variable. In the specific context of this study, it necessitates that no systematic differences exist between the trends of emergency logistics response capacity in the treatment group and those in the control group before the implementation of emergency logistics policies. Should the parallel trend assumption be violated, DID estimation results may be confounded by inherent inter-group disparities, thereby precluding the accurate identification of the causal effect of the policy.

**Figure 2:**
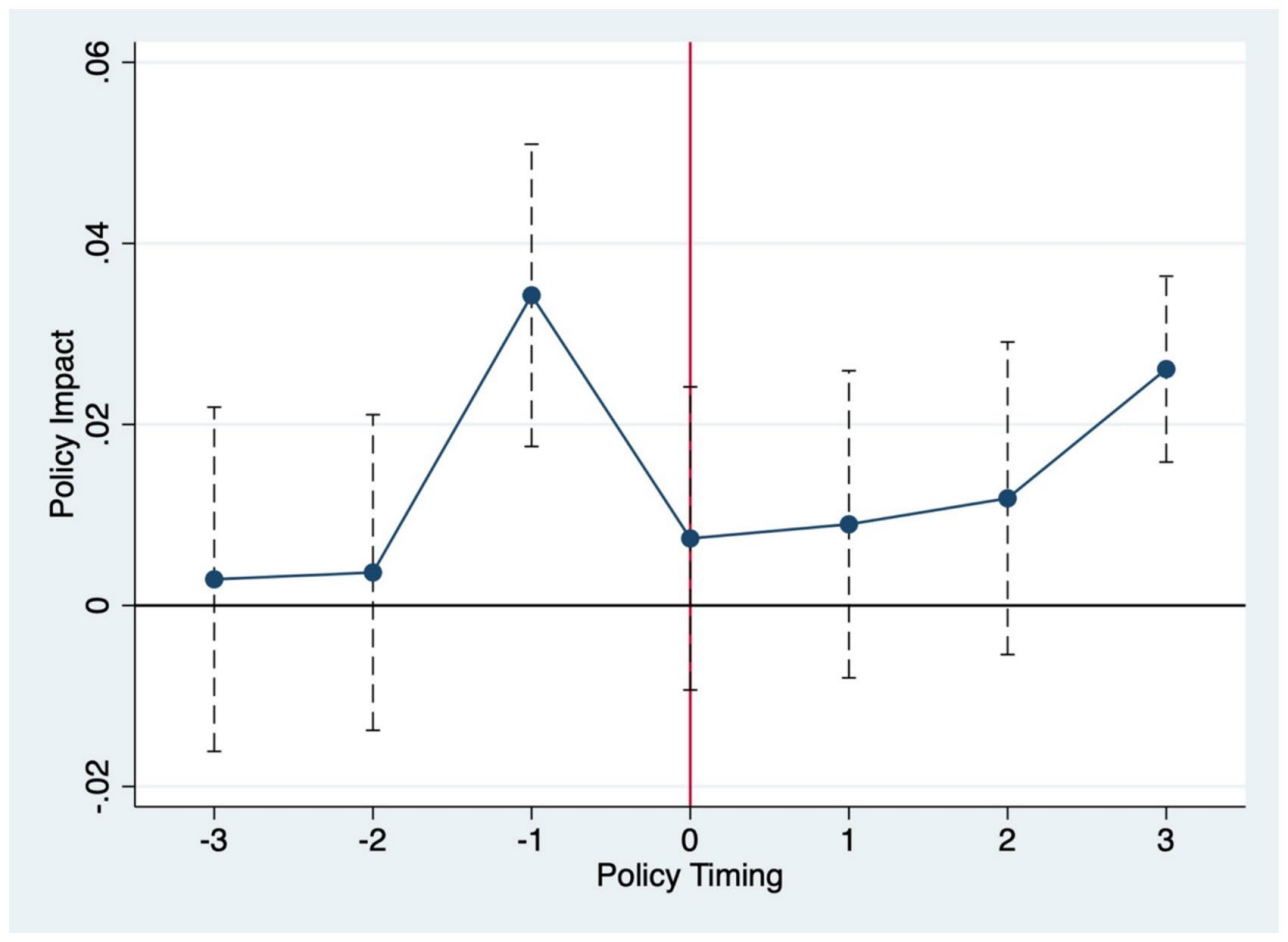
Parallel trend test results.

The test results are presented in Figure 2. The 95% confidence intervals for the interaction term coefficients of all pre-policy periods—namely pre_3 to pre_1—encompass zero, indicating that prior to policy implementation, no significant difference existed between the trends of emergency logistics response capacity in the treatment group and those in the control group. Thus, the parallel trend assumption is satisfied.

With respect to the dynamic effects following policy implementation, the interaction term coefficients for the post_1 and post_2 periods are statistically insignificant, whereas the coefficient for the post_3 period is significantly positive, with its confidence interval lying entirely above zero. This finding suggests that emergency logistics policies do not take effect immediately upon promulgation; rather, the release of policy effects entails an “institutional bedding-in period” of approximately 2 years. During this interval, local governments must complete preliminary preparations encompassing institutional restructuring, resource integration, personnel training, and information system alignment. Concurrently, activities such as the construction of emergency material reserve warehouses, the signing of emergency agreements with logistics enterprises, and the establishment of cross-regional coordination mechanisms require time for gradual implementation. It is only by the third year following policy implementation that these institutional arrangements begin to translate into observable capacity enhancements. This discovery substantiates the positive impact of policies on emergency logistics response capacity while simultaneously revealing the time-lag characteristic inherent in the realization of policy efficacy.

### 5.3 Robustness checks

To ensure the robustness of the baseline regression results, this paper conducts robustness checks from the following aspects.

(1) Excluding observations prior to 2016.

Column (1) of Table 11 reports the regression results based on the sample retaining only observations from 2016 onward. The year 2016 is chosen as the cutoff point because the National Comprehensive Disaster Prevention and Mitigation Plan (2016–2020) was implemented in that year, marking the entry of China’s emergency management policy system into a refinement stage, with a greater emphasis on building disaster prevention, mitigation, and relief capabilities. Excluding observations prior to 2016 facilitates a more precise examination of the impact of recent policies on emergency logistics response capacity.

**Table 11:** Robustness checks.

	(1) Shortened sample period	(2) Exclusion of directly administered municipalities
	C	C
DID	0.0174^***^	0.0148^**^
	(2.9131)	(2.4079)
Lnforest	0.0000	0.0000
	(0.8841)	(0.5620)
Lnarea	0.0000	−0.0000
	(0.6887)	(−0.8640)
Lndisaster	0.0000	−0.0000
	(0.5199)	(−0.9505)
Lngdp	0.0149^***^	0.0058
	(3.3276)	(1.6510)
_cons	−0.1314^***^	−0.0414
	(−2.8757)	(−1.1150)
N	210	468
F	3.9458^***^	2.9677^**^
r2_a	0.2207	0.0105

t statistics in parentheses. *p < 0.1, **p < 0.05, ***p < 0.01.

The regression results show that the DID coefficient is 0.0174, statistically significant at the 1% level. Using the sample mean of emergency logistics response capacity (0.0292) as a reference, this coefficient implies that provinces implementing the policy exhibited approximately 60% higher response capacity than those that did not. Compared with the baseline regression coefficient of 0.0137, this estimate has increased. The economic implication of this change is that, in the policy refinement stage after 2016, although the room for marginal improvement may theoretically have narrowed, the estimated coefficient has actually become larger. This reflects that later-stage policies became more precise and effective in targeting objectives, resource mobilization, and cross-departmental coordination, thereby unleashing greater capacity gains. In other words, as the institutional design of policies continues to improve, their implementation effects have not attenuated over time. This result is consistent with the baseline regression findings, indicating that the facilitating effect of emergency logistics policies remains robust after adjusting the sample time frame.

(2) Excluding samples from centrally administered municipalities.

Column (2) of Table 11 reports the regression results after excluding the four municipalities directly under the central government—Beijing, Shanghai, Tianjin, and Chongqing. As megacities with highly concentrated economic and administrative resources, these municipalities typically exhibit substantially higher emergency logistics capacity than other regions; retaining these samples could overestimate the overall policy effect. Excluding them allows for a more accurate assessment of the actual effect of emergency logistics policies in ordinary provincial-level regions.

The regression results show that the DID coefficient is 0.0148, significant at the 5% level, corresponding to a capacity improvement of approximately 51%, slightly higher than the baseline regression estimate of 0.0137. The implication of this result is that, even after excluding the potential overestimation factor posed by municipalities, the net effect of emergency logistics policies remains robust and does not decline. From an institutional perspective, municipalities typically possess more abundant fiscal resources and more efficient administrative systems, and their emergency logistics capacity is already at a relatively high level; after their exclusion, the policy effect in ordinary provinces actually increases slightly, indicating that the policy can also play a significant role in regions with relatively limited resources and may even exhibit a stronger relative effect due to the higher marginal benefit of policy intervention. This test rules out the possibility that the baseline regression results are driven by the sample of municipalities.

(3) Placebo test.

**Figure 3:**
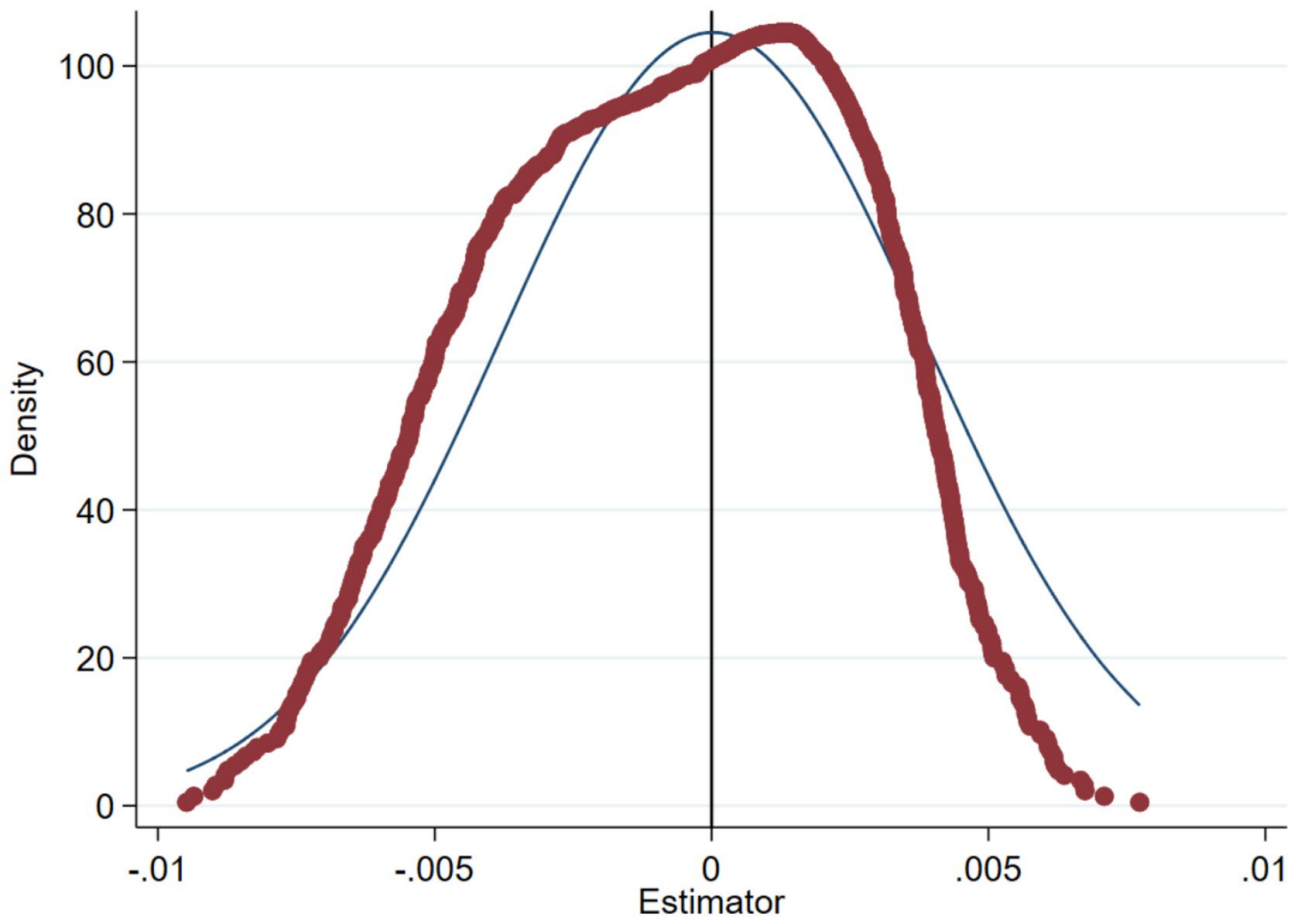
Placebo test results.

To verify whether the baseline difference-in-differences (DID) estimation results are driven by chance factors or unobservable random disturbances, this paper conducts a placebo test by randomly permuting the treatment group assignment. Specifically, we randomly assign treatment group status across the full sample and repeatedly estimate the baseline DID model, thereby obtaining a series of placebo estimated coefficients and their empirical distribution. The results are presented in Figure 3, where the red curve represents the distribution of DID coefficients obtained after random assignment of the treatment group, and the blue curve represents the corresponding theoretical normal distribution curve. It can be clearly observed that the placebo estimated coefficients are highly concentrated around zero, with a mean close to zero, and the shape of the coefficient distribution fits the theoretical normal distribution well, exhibiting no systematic deviation or obvious bias. This result indicates that the treatment effect estimated in the baseline regression is fundamentally distinct from effects generated by random factors and is unlikely to be attributable to other stochastic factors, thereby further strengthening the robustness and causal identification validity of the core findings of this paper.

### 5.4 Heterogeneity analysis

#### 5.4.1 By geographic region

The preceding measurement results reveal that China’s regional emergency logistics response capacity exhibits a gradient distribution of “higher in the east and lower in the west”: the mean value in the eastern region (0.03573) is higher than that in the central (0.03417) and western regions (0.01854), and the growth rate in the eastern region (6.07%) also exceeds those of the central (3.39%) and western regions (4.15%). Meanwhile, the measurement of policy coherence indicates that eastern provinces include both high-coherence regions such as Guangdong and Shandong and low-coherence regions such as Beijing and Jiangsu, whereas central and western provinces exhibit a coexistence of high, medium, and low levels of coherence, with overall coherence slightly lower than that of the eastern region. Correlation analysis between policy coherence and response capacity further suggests that high policy coherence does not necessarily lead to high response capacity, but low coherence is generally accompanied by low capacity. These findings suggest that policy effects may vary depending on regional conditions such as economic foundation, infrastructure, and informatization levels. To empirically test this inference, this study divides the sample into the eastern region and the central and western regions for separate regression analyses, following the regional classification standard of the National Development and Reform Commission.

The subgroup regression results are reported in Table 12. In the eastern region, the DID coefficient is 0.0187, statistically significant at the 5% level. Using the sample mean of emergency logistics response capacity (0.0292) as a reference, this implies that policy implementation increased the emergency logistics capacity of eastern provinces by approximately 64%. The eastern region possesses a more developed transportation network, higher levels of informatization, and a more mature market-oriented logistics system, enabling policies to be rapidly translated into actual emergency response capacity once introduced. This effect magnitude is notably higher than the medium-term effect of emergency policies observed in developed countries, approximately 35%, reported in existing studies, which is inseparable from the institutional execution capacity and resource integration ability accumulated by China’s eastern region as the pioneer area of reform and opening-up. In the central and western regions, the DID coefficient is 0.0069, but it fails to pass the significance test. In terms of the coefficient magnitude, if the effect were to exist, the corresponding capacity improvement would be approximately 23.6%, less than half of that in the eastern region. The central and western regions have relatively weak logistics infrastructure, insufficient informatization, and limited fiscal resources, which create additional obstacles in the process of translating policies from textual provisions into practical implementation. From an institutional perspective, government effectiveness is the most stable and policy-sensitive predictor of logistics performance, and the gap in policy implementation quality in the central and western regions precisely explains the lack of policy effects. The above results confirm the dependence of policy effectiveness on regional endowments and further elucidate the previously observed phenomenon that “high policy coherence does not necessarily lead to high response capacity”—even if certain central and western provinces have policy texts highly consistent with central planning, the lack of infrastructure and information support comparable to that in the eastern region makes it difficult for policy effects to materialize. This implies that the optimization of emergency logistics policies should not remain merely at the level of textual alignment but must be advanced in synergy with substantive regional capacity building.

**Table 12:** Heterogeneity analysis (I).

	(1) East China	(2) Central and Western China
	C	C
DID	0.0187^**^	0.0069
	(2.2480)	(1.2655)
Lnforest	−0.0000	0.0070
	(−0.0475)	(0.8336)
Lnarea	−0.0000	−0.0000
	(−0.6878)	(−0.6874)
Lndisaster	0.0000	−0.0000
	(0.2065)	(−0.7412)
Lngdp	−0.0019	0.0016
	(−0.3731)	(0.3859)
_cons	0.0422	0.0041
	(0.7039)	(0.1049)
*N*	216	324
*F*	4.3153**	1.6985
r2_a	0.1597	−0.0102

t statistics in parentheses. ^*^p < 0.1, ^**^p < 0.05, ^***^p < 0.01.

#### 5.4.2 By policy consistency

The preceding analysis measured the textual coherence between provincial emergency policies and the central master plan, grouping provinces with policy coherence scores ≥ 0.75 into the high-coherence group and the remaining into the low-coherence group. The subgroup regression results are presented in Table 13. In the high-coherence group (*N* = 126), the DID coefficient is 0.0202, significant at the 1% level; in the low-coherence group (*N* = 414), the DID coefficient is 0.0117, significant at the 5% level. Both coefficients are positive and statistically significant, indicating that the implementation of emergency logistics policies can enhance response capacity regardless of the level of policy coherence.

**Table 13:** Heterogeneity analysis (II).

	(1) High policy consistency	(2) Low policy consistency
	C	C
DID	0.0202***	0.0117**
	(4.0233)	(2.2563)
Lnforest	0.0000**	0.0000
	(2.2024)	(0.4882)
Lnarea	−0.0000	−0.0000
	(−0.7538)	(−0.2908)
Lndisaster	−0.0000	−0.0000
	(−0.4951)	(−0.3144)
Lngdp	−0.0004	0.0046
	(−0.0985)	(1.3282)
_cons	0.0223	−0.0286
	(0.5158)	(−0.7643)
*N*	126	414
*F*	4.6854	1.2008
r2_a	0.1285	0.0024

t statistics in parentheses. ^*^p < 0.1, ^**^p < 0.05, ^***^p < 0.01.

In terms of coefficient magnitude, the policy effect in the high-coherence group (0.0202) is approximately 1.73 times that in the low-coherence group (0.0117). Using the sample mean of 0.0292 as a reference, the improvement in response capacity after policy implementation is approximately 69.2% in the high-coherence group and 40.1% in the low-coherence group. The economic implication of this gap is that the more a province’s policy text aligns with the central top-level design in terms of discourse structure, goal setting, and mechanism design, the more readily policy implementation translates into actual capacity gains. In other words, policy coherence functions as an “institutional amplifier.” This finding aligns with the assertion in policy mix design theory that coordination is a key determinant of policy effectiveness—when local policies converge highly with the central framework at the semantic level, the synergistic efficiency of resource mobilization and response speed are significantly enhanced.

It is worth noting that the policy effect in the low-coherence group remains significant, indicating that even when local policies deviate considerably from central planning, policy implementation itself can still yield a certain degree of capacity improvement. This result is not contradictory to the earlier observation that “high policy coherence does not necessarily lead to high emergency logistics performance.” Coherence serves as a “facilitating factor” rather than a “determining factor” for policy effectiveness: some provinces in the low-coherence group may have achieved capacity growth through alternative pathways such as local independent innovation or infrastructure construction, yet their average policy effect is significantly lower than that of the high-coherence group. From an institutional perspective, this disparity reflects the critical role of coordination efficiency in system resilience—when policy interventions lack coherence across different governance levels, their effectiveness is diminished.

#### 5.4.3 By initial level of emergency logistics response capacity

The preceding measurement results show substantial variation in the initial emergency logistics response capacity across provinces, with provinces such as Hubei, Guangdong, and Shandong exhibiting relatively high initial capacity, while provinces such as Qinghai, Ningxia, and Gansu exhibit relatively low initial capacity. To examine whether the policy effect varies with the initial capacity level, this paper uses the emergency logistics response capacity in the year prior to policy implementation as the baseline initial level and divides the sample into a high initial capacity group and a low initial capacity group by the median, conducting separate regression analyses for each.

The subgroup regression results are presented in Table 14. In the low initial capacity group (column 1), the DID coefficient is 0.0139, statistically significant at the 10% level; in the high initial capacity group (column 2), the DID coefficient is 0.0171, statistically significant at the 1% level. Both coefficients are positive and statistically significant, indicating that the implementation of emergency logistics policies can enhance response capacity regardless of the initial capacity level.

**Table 14:** Heterogeneity analysis (III).

	(1) Low initial level	(2) High initial level
	C	C
DID	0.0139*	0.0171***
	(1.7622)	(8.4740)
Lnforest	0.0000	−0.0000
	(1.5794)	(−0.9146)
Lnarea	−0.0000*	0.0000
	(−1.8014)	(1.0896)
Lndisaster	−0.0000	−0.0000
	(−0.6845)	(−0.9342)
Lngdp	0.0027	0.0047***
	(0.4307)	(3.8899)
_cons	0.0035	−0.0413***
	(0.0514)	(−3.1295)
N	270	270
F	1.4973	16.0771
r2_a	0.0092	0.2189

t statistics in parentheses. *p < 0.1, **p < 0.05, ***p < 0.01.

In terms of coefficient magnitude, the policy effect in the high initial capacity group (0.0171) exceeds that in the low initial capacity group (0.0139). Using the sample mean of 0.0292 as a reference, the improvement in response capacity after policy implementation is approximately 58.6% in the high initial capacity group and 47.6% in the low initial capacity group. This gap reflects an “amplifier effect” of initial capacity on policy outcomes: regions with well-developed infrastructure and advanced logistics systems can more rapidly convert policy directives into actual capacity gains, whereas regions with a weak foundation, though also benefiting from the policy, experience relatively limited improvement. From the perspective of system resilience theory, the absorptive and adaptive capacities of a system constitute core dimensions of emergency response, and the results of this paper indicate that such initial capacity also moderates the effectiveness of policy interventions. At the same time, this result corroborates the earlier finding that “the growth rate in the eastern region is higher than that in the central and western regions,” further demonstrating that the release of emergency logistics policy effectiveness depends on the existing capacity foundation of the region.

It is worth noting that the policy effect in the low initial capacity group remains statistically significant, suggesting that emergency logistics policies serve not only to “reinforce existing strengths” but also to “remedy critical deficiencies.” For regions with a weak foundation, the institutional driving force provided by policies can, to some extent, compensate for inadequate endowments; however, this compensatory effect needs to be consolidated through sustained infrastructure investment and capacity cultivation. From a policy implication perspective, this means that when implementing emergency logistics policies in low-capacity regions, they should be accompanied by capacity-building programs rather than relying solely on the dissemination of policy texts.

### 5.5 Analysis of the moderating effect of policy consistency

According to the measurement results presented in Section 3, the textual consistency between provincial emergency policies and the central contingency plan exhibits substantial variation across provinces. Specifically, provinces such as Chongqing, Jilin, Guangdong, Shaanxi, Inner Mongolia, and Henan demonstrate relatively high consistency (≥0.75), whereas provinces such as Tianjin, Shanxi, and Qinghai exhibit relatively low consistency. To examine whether policy consistency moderates the implementation effect of emergency logistics policies, this study introduces a dummy variable for high policy consistency and its interaction term with *DID* into the baseline model (11), thereby constructing the following moderating effect model shown in Equation 12:


$$\begin{array}{l} c_{i,t}=\alpha_{0}+\alpha_{1}\mathrm{DI}D_{\mathrm{it}}+\alpha_{2}\text{Infores}t_{\mathrm{it}}+\alpha_{3}\text{Inare}a_{\mathrm{it}}+\\ \alpha_{4}\text{Indisaste}r_{\mathrm{it}}+\alpha_{5}\text{Ingd}p_{\mathrm{it}}+\alpha_{6}\text{high}\_\mathrm{con}s_{i}+\\ \alpha_{7}(\mathrm{DI}D_{\mathrm{it}}\times \mathrm{hig}h_{\text{cons}i})+\varepsilon_{\mathrm{it}} \end{array}$$ (12)

Wherein, *high_cons_i_* denotes a dummy variable for high policy consistency, taking the value of 1 when policy consistency ≥ 0.75, and 0 otherwise. $\mathrm{DI}D_{\mathrm{it}}\times \text{high}\_\mathrm{con}s_{i}$ represents the interaction term, denoted in the table as DID_high, which captures the incremental policy effect of the high policy consistency group relative to the low consistency group. A significantly positive coefficient for this term would indicate that policy consistency positively moderates the policy effect. All other variables remain identical to those specified in the baseline model above.

Column (1) presents the baseline model, with a DID coefficient of 0.0137, statistically significant at the 5% level. In column (2), after adding the interaction term, the DID coefficient decreases to 0.0115 and its significance level drops to 10%; the coefficient of the interaction term DID_high is 0.0092, but it fails to pass the significance test. In terms of coefficient magnitude, the policy effect in the high-coherence group is approximately 1.80 times that in the low-coherence group, a ratio highly close to the 1.73 times found in the subgroup regression. Using the sample mean of emergency logistics response capacity (0.0292) as a reference, the improvement in response capacity after policy implementation is approximately 39.4% in the low-coherence group and 70.9% in the high-coherence group. This means that in provinces where policy texts are highly consistent with central planning, the capacity gain brought by the policy is approximately 31.5 percentage points higher than in low-coherence provinces. From an institutional perspective, this gap indicates that policy coherence indeed affects the extent to which policy effectiveness is unleashed: when local policies converge with the central framework in goal setting and mechanism design, the efficiency with which policies are translated from text into actual capacity is higher. This is consistent with prior research concluding that policy coordination is a key determinant of policy effectiveness.

However, the interaction term fails to attain statistical significance, indicating that the difference in policy effects between the high-coherence and low-coherence groups is not statistically robust. Several reasons may account for this result. First, the sample size of the high-coherence group is only 126, while that of the low-coherence group is 414; this substantial disparity in sample size leads to lower estimation precision and limited statistical power for the high-coherence group. Second, the full-sample interaction model employs province-clustered robust standard errors, a relatively stringent treatment that further raises the threshold for statistical significance. More importantly, the moderating effect of policy coherence may not operate as a simple threshold effect—that is, it does not necessarily become clearly manifest once the 0.75 cutoff is exceeded. In practice, the influence of policy coherence on policy effectiveness may be jointly constrained by confounding factors such as local implementation capacity, infrastructure levels, and fiscal resources. The effectiveness of policy interventions across different governance levels depends on their coordination efficiency, and coordination efficiency itself is a multidimensional concept, of which mere textual policy coherence constitutes only one component. Furthermore, the fact that in the subgroup regressions both coefficients are statistically significant and the coefficient in the high-coherence group is larger still indicates that policy coherence exerts a positive facilitating effect on policy effectiveness. Therefore, Hypothesis H2 of this study is supported.

Taken together, although the interaction term does not reach statistical significance, the significant difference in coefficients between the two groups in the subgroup regressions, along with the magnitude of the coefficient ratio, suggests that policy coherence remains an important factor influencing the effectiveness of emergency logistics policies. The policy implication of this finding is that enhancing the coherence between local emergency policies and central planning helps strengthen the actual effects of policy interventions; however, improving coherence should go beyond mere textual alignment and be advanced in synergy with complementary conditions such as implementation capacity and infrastructure (Table 15).

**Table 15:** Analysis of the moderating effect of policy consistency.

	(1)	(2)
	Baseline model	Moderating effect model
DID	0.0137**	0.0115*
	(2.4431)	(1.7926)
Lnforest	0.0000	0.0000
	(0.6604)	(0.6534)
Lnarea	−0.0000	−0.0000
	(−0.8469)	(−0.8041)
Lndisaster	−0.0000	−0.0000
	(−0.9025)	(−0.8623)
Lngdp	0.0036	0.0038
	(1.2497)	(1.4000)
DID_high		0.0092
		(0.8984)
high_cons		0.0010
		(0.1249)
_cons	−0.0181	−0.0204
	(−0.5826)	(−0.7080)
N	540	540
r2_a	0.0121	0.0158

t statistics in parentheses. *p < 0.1, **p < 0.05, ***p < 0.01.

## 6 Discussion

Taking the promulgation of China’s National Master Plan for Public Emergencies in 2006 as a quasi-natural experiment, this paper uses panel data from 30 provincial-level administrative regions spanning 2005 to 2022 and employs a staggered difference-in-differences approach to systematically evaluate the impact of emergency logistics policies (emergency contingency plans) on emergency logistics response capacity. Unlike existing studies that primarily focus on route planning or material allocation algorithms in emergency logistics, this paper adopts an institutional evaluation perspective, extending the scope of emergency logistics research from the technical question of “how to do better” to the institutional question of “whether policies are effective,” thereby filling the gap in the existing literature regarding the lack of systematic causal inference on emergency logistics policies themselves.

First, this paper employs a cosine similarity-based text quantification method to systematically analyze the deep semantic structure of provincial emergency logistics policies. This approach goes beyond the conventional practice in traditional policy text analysis of relying on simple statistical indicators such as keyword frequency or policy intensity, and instead systematically characterizes the convergence and divergence of policies across regions at the semantic level, thereby introducing the dimension of “policy content differences”—long neglected in empirical research—into the empirical model. The measurement results show that the national average policy coherence is approximately 0.53, with significant regional divergence. Further analysis reveals that low policy coherence is generally accompanied by low emergency logistics response capacity, whereas high policy coherence does not directly determine high response capacity. This finding complements the existing literature’s assertion that policy coordination is a key determinant of policy effectiveness: while prior research emphasizes the importance of coordination per se, our results indicate that policy coherence serves more as a fundamental supporting condition rather than a sufficient condition. In other words, relying solely on the consistency of policy content is insufficient to translate it directly into actual capacity gains; it requires the synergistic support of factors such as infrastructure, informatization level, and implementation capacity. This finding reveals the boundary conditions of the role of policy coherence, thereby refining and extending the theory of policy effectiveness.

Second, in terms of measuring emergency logistics response capacity, this paper constructs a multidimensional composite indicator system encompassing carrier carrying capacity, potential circulation capacity, potential velocity capacity, potential efficiency capacity, regional informatization capacity, and social emergency support capacity, and employs the entropy-weighted TOPSIS method to measure the emergency logistics response capacity of 30 Chinese provinces from 2005 to 2022. The results show that China’s overall emergency logistics response capacity exhibits an upward trend, yet the regional disparity continues to widen: the mean value in the eastern region is significantly higher than that in the central and western regions, and the growth rate in the eastern region likewise exceeds those of the central and western regions. This finding provides quantitative evidence for understanding the unbalanced spatial pattern of “higher in the east and lower in the west” in China’s emergency logistics capacity and fills a gap in existing research, which has largely focused on the key factors affecting emergency logistics while overlooking the temporal dynamics and regional differences of emergency logistics response capacity itself. Compared with the medium-term effect of emergency policies observed in developed countries in cross-national studies (approximately 35%), the capacity improvement of 47% in the baseline regression of this paper is at a relatively high level, reflecting China’s institutional implementation advantages as a late-developing country in building its emergency management system.

Finally, by constructing a staggered difference-in-differences model and leveraging the institutional context in which provinces implemented emergency logistics policies in a phased manner, this paper achieves a quasi-experimental causal inference of policy effects and systematically examines the dynamic effects and heterogeneous patterns. The empirical results show that the enhancement of response capacity fails to reach statistical significance in the first 2 years after policy implementation, emerging only from the third year onward and continuing to strengthen thereafter. This finding indicates that the improvement of emergency logistics response capacity is not an immediate reaction to policy promulgation; rather, it depends on a gradual process involving institutional building, resource integration, and capacity cultivation. This time-lag characteristic is consistent with observations in existing research regarding the “institutional adjustment period” in policy implementation, and this paper further quantifies the specific duration of this lag (approximately 2 years), providing policymakers with a more precise basis for expectation management. In terms of heterogeneity, the policy effect exhibits significant regional divergence: it is significant in the eastern region but not in the central and western regions, which corroborates, from the reverse side, the established finding that government effectiveness is the most stable and policy-sensitive predictor of logistics performance. Subgroup analysis based on policy coherence shows that the policy effect in the high-coherence group is higher than that in the low-coherence group, revealing the positive moderating role of institutional alignment on policy implementation outcomes. Subgroup analysis based on initial capacity indicates that the policy can serve a compensatory function of “remedying critical deficiencies” in regions with a weak foundation, while generating an incremental enhancement effect of “reinforcing existing strengths” in regions with a sound foundation. This mechanism analysis deepens the understanding of the conditions under which emergency policy effectiveness is realized and highlights the importance of formulating differentiated emergency management policies.

From a global perspective, China, as the largest developing country, exhibits a “gradualist” pattern in its emergency policy implementation—characterized by a phased, province-by-province rollout with pilot programs taking the lead. This provides a natural experimental setting for the staggered difference-in-differences method, in contrast to the “nationwide synchronized implementation” model typical of Western countries. The empirical findings of this paper reveal a core proposition: the institutional advantages of emergency contingency plans can only be effectively realized when underpinned by sound logistics infrastructure and a high level of informatization. This conclusion not only offers reference experience for other developing countries that similarly face pronounced regional development disparities and high-frequency disaster risks, but also provides a comparative case for developed countries to understand the interplay between institutional design and infrastructure synergy in emergency management.

## 7 Conclusion and recommendations

### 7.1 Research conclusion

Enhancing emergency logistics response capacity constitutes a critical measure for improving the national emergency management system and safeguarding public safety, property, and social stability. Utilizing panel data from 30 provincial-level administrative regions in China spanning the period 2005–2022, and taking the promulgation of the National Master Plan for Public Health Emergency Response in 2006 as the policy backdrop, this study employs a staggered difference-in-differences approach to systematically examine the impact of emergency policies (contingency plans) on emergency logistics response capacity. The principal research conclusions are as follows:

First, emergency policy coherence provides foundational support for regional emergency logistics response capacity but does not constitute a sufficient condition. Using a cosine similarity-based text quantification method to measure the coherence between provincial emergency policies and the central master emergency plan, we find that the national average policy coherence is approximately 0.53, with significant regional divergence. Provinces simultaneously exhibiting low policy coherence and low emergency logistics response capacity account for 40% of the total sample, indicating that deviation of policy texts from the national framework is generally accompanied by insufficient capacity. However, provinces simultaneously displaying high coherence and high response capacity constitute only 20% of the sample; some high-coherence provinces, such as Chongqing, Jilin, and Inner Mongolia, still fall within the medium-to-low capacity range. This finding suggests that policy text alignment is a necessary foundation for enhancing emergency logistics response capacity, yet coherence alone is insufficient to directly translate into actual capacity gains; it also requires the synergistic support of factors such as infrastructure, informatization level, and implementation capacity. This quantitative conclusion advances the theory of policy coherence from qualitative discussion to a testable empirical level, reveals the boundary conditions of its role, and constitutes one of the theoretical contributions of this paper.

Second, the implementation of emergency policies significantly enhances regional emergency logistics response capacity. The baseline regression results show that, after controlling for the number of forest fires, total burned area, the number of geological disasters, and the level of regional economic development, the estimated policy effect coefficient is 0.0137, statistically significant at the 5% level. Using the sample mean of emergency logistics response capacity as a reference, provinces that implemented the policy exhibited approximately 47% higher response capacity than those that did not. This result indicates that emergency logistics policies not only possess statistical significance but also yield substantial capacity gains in actual emergency response. This conclusion withstands multiple robustness checks, demonstrating that the policy effect is stable and reliable. Based on Chinese provincial panel data, this finding provides large-sample causal inference evidence on the effects of emergency logistics policies, filling the gap in existing research that is largely confined to case studies or cross-national comparisons, and constitutes the core empirical contribution of this paper.

Third, the effect of emergency policies exhibits a significant time lag, and the release of policy effectiveness requires a gradual process. The parallel trend test results show that the coefficients of the interaction terms for all pre-policy periods are statistically insignificant, satisfying the parallel trend assumption. In the first and second years after policy implementation, the policy effect had not yet materialized; it became significantly positive only from the third year onward. This time-lag phenomenon reflects the inherent logic of emergency logistics response capacity improvement: after policy promulgation, local governments need to complete preparatory work such as institutional restructuring, resource integration, personnel training, and information system alignment. Meanwhile, activities such as constructing emergency material reserve warehouses, signing emergency agreements with logistics enterprises, and establishing cross-regional coordination mechanisms all require time for gradual implementation. It is only around the third year after policy implementation that these institutional arrangements begin to translate into observable capacity improvements. This provides empirical evidence for understanding the “institutional bedding-in period” from policy promulgation to effectiveness, enriching the discussion on the temporal dimension in policy implementation theory.

Fourth, the policy effect exhibits significant regional heterogeneity, and the release of policy effectiveness depends on the synergistic coordination of multiple conditions. From a geographical perspective, the policy effect is significant in the eastern region but not in the central and western regions, indicating that the eastern region, with its more developed transportation network and higher level of informatization, can more rapidly translate policy into capacity. From the perspective of policy coherence, the policy effect in the high-coherence group is significantly larger than that in the low-coherence group, reflecting a “coherence dividend” generated by institutional alignment. From the perspective of initial capacity, the policy effect in the high initial capacity group is stronger than that in the low initial capacity group, and both are statistically significant, demonstrating that the policy can not only “remedy critical deficiencies” for regions with a weak foundation but also “reinforce existing strengths” for regions with a sound foundation. These three dimensions of heterogeneity are interrelated: geographical region determines the baseline level of infrastructure, policy coherence reflects the degree of institutional alignment, and initial capacity embodies long-term endowment accumulation. Collectively, these findings indicate that the effectiveness of emergency logistics policies is highly dependent on the synergistic coordination of regional infrastructure, informatization level, institutional alignment, and initial capacity. This mechanism analysis deepens the understanding of the conditions under which emergency policy effectiveness is realized and provides an empirical basis for designing differentiated policies.

### 7.2 Policy recommendations

Based on the aforementioned research conclusions and considering the practical demands of emergency logistics development in China, this paper proposes the following policy recommendations:

First, building on policy coherence, infrastructure and implementation capacity should be advanced in parallel. This paper finds that policy coherence is a necessary foundation for enhancing emergency logistics response capacity, yet it is not a sufficient condition; high policy coherence does not necessarily lead to high response capacity, whereas low coherence is generally accompanied by low capacity. Therefore, when revising emergency plans, regional authorities should use the national master emergency plan as a benchmark to ensure standardized alignment of core objectives, key mechanisms, and operational procedures, while allowing appropriate local adaptation and innovation. More importantly, investment in emergency logistics infrastructure in the central and western regions must be simultaneously scaled up, including the construction of regional emergency material reserve centers, the upgrading of transportation networks, and the improvement of emergency command information systems. This is to ensure that the “alignment” of policy texts can genuinely translate into capacity gains, thereby avoiding the dilemma of “high coherence but low capacity.”

Second, the time-lag characteristic of policy effects should be respected, and a long-term, stable mechanism for policy implementation and evaluation should be established. This paper finds that the effect of emergency policies becomes significantly manifest only in the third year after implementation. This implies that policymakers should maintain strategic patience and avoid prematurely adjusting or terminating policies due to insignificant short-term effects. It is recommended that a phased evaluation mechanism be established: in the initial implementation phase (the first 2 years), the focus should be on examining institutional building and resource integration to ensure that foundational work such as institutional restructuring, personnel training, and information system alignment is in place; in the medium-term implementation phase (years three to five), the emphasis should be on evaluating capacity enhancement and operational efficiency; in the long term (beyond 5 years), a comprehensive assessment of overall effectiveness release should be conducted. Meanwhile, for emergency policies that have been demonstrated to significantly enhance response capacity, efforts should be made to further promote their full coverage and eliminate policy blind spots.

Third, adopt differentiated regional policies tailored to local conditions. For the central and western regions, where the policy effect has yet to materialize, it is necessary to increase investment in emergency logistics infrastructure through mechanisms such as central fiscal transfers and special bonds, and to establish paired assistance mechanisms between the eastern region and the central and western regions to gradually narrow the regional capacity gap. For regions with low policy coherence, standardized policy text templates should be developed, clearly delineating the boundary between core elements and locally distinctive content, and a dynamic revision mechanism should be established to periodically assess the alignment between coherence and actual outcomes; meanwhile, mutual recognition and alignment of policy texts across provinces should be promoted to lay an institutional foundation for regional emergency coordination. For regions with low initial capacity, although the policy effect is significant, its magnitude is moderate; policy implementation should be accompanied by capacity-building programs and sustained investment to consolidate and amplify the policy dividend, so as to prevent capacity from regressing due to interrupted investment. For regions with high initial capacity, their institutional implementation advantages should be fully leveraged, and pioneering trials should be encouraged, thereby providing replicable experience models for the rest of the country.

Fourth, strengthen policy standardization and coherence to enhance the synergy between central and local policies. It is recommended that a coordination mechanism for central-local emergency policy formulation be established, with regular policy coherence reviews. Meanwhile, the improvement of policy coherence should not remain at the level of mere textual alignment but must be advanced in synergy with complementary conditions such as implementation capacity and infrastructure. Specifically, a cross-level policy coordination committee could be established to review the coherence between provincial emergency plans and the national master plan and to provide revision guidance.

Fifth, based on the time-lag characteristic, the timing of policy implementation should be optimized. It is recommended that in the central and western regions and regions with low initial capacity, institutional building and capacity cultivation be initiated in advance, so that these regions already possess the necessary institutional foundation and absorptive capacity when the policy is formally implemented, thereby shortening the time lag of policy effects. Specifically, a one- to two-year “capacity cultivation preparatory period” could be established prior to the formal implementation of the policy, during which priority is given to completing preparatory work such as institutional setup, personnel training, and information system construction.

### 7.3 Research limitations and future directions

This study is subject to several limitations that warrant further investigation in future research. First, the measurement of policy consistency is based on the cosine similarity between provincial policy texts and the central contingency plan and does not yet incorporate variations at the policy implementation level. Future research could integrate field investigations or implementation data to examine the impact of “text–implementation” discrepancies on policy effectiveness. Second, while this study constructs a multidimensional indicator system for emergency logistics response capacity, the specific contributions and dynamic interactions of its sub-dimensions—such as carrier carrying capacity, flow volume capacity, flow velocity capacity, flow efficiency capacity, regional informatization capacity, and social emergency support capacity—remain underexplored. Subsequent research could disaggregate these sub-dimensions to examine how each responds differently to policy interventions and to identify potential trade-offs or synergies among them. Third, the selection of control variables in this study, though grounded in the existing literature, may not fully account for all confounding factors affecting emergency logistics response capacity. Future research should expand the set of control variables to include more refined measures of institutional quality, local fiscal capacity, and geographic vulnerability, thereby enhancing the precision of policy effect estimation. Furthermore, the measurement of emergency logistics response capacity relies on publicly available statistical data, which may not fully capture the real-time dynamics and synergistic aspects of emergency response. Subsequent studies could incorporate high-frequency data or comparative case analyses for supplementary validation. Overall, the aforementioned limitations delineate clear directions for future research. In-depth exploration of these issues will contribute to a more comprehensive understanding of the mechanisms through which emergency logistics policy efficacy is realized.

## Data Availability

The raw data supporting the conclusions of this article will be made available by the authors, without undue reservation.
